# A Gene–Environment Interaction Between Smoking and Gene polymorphisms Provides a High Risk of Two Subgroups of Sarcoidosis

**DOI:** 10.1038/s41598-019-54612-1

**Published:** 2019-12-09

**Authors:** Natalia V. Rivera, Karina Patasova, Susanna Kullberg, Lina Marcela Diaz-Gallo, Tomoko Iseda, Camilla Bengtsson, Lars Alfredsson, Anders Eklund, Ingrid Kockum, Johan Grunewald, Leonid Padyukov

**Affiliations:** 1 Division of Respiratory Medicine, Department of Medicine Solna, Karolinska Institutet, Karolinska University Hospital, SE-171 76 Stockholm, Sweden; 2 Division of Rheumatology, Department of Medicine Solna, Karolinska Institutet, Karolinska University Hospital, SE-171 76 Stockholm, Sweden; 3 Institute of Environmental Medicine (IMM), Karolinska Institutet, SE-171 77 Stockholm, Sweden; 4 Neuroimmunology Unit, Department of Clinical Neuroscience, Karolinska Institutet, SE-171 76 Stockholm, Sweden

**Keywords:** Disease genetics, Translational research

## Abstract

The influence and effect of cigarette smoking in sarcoidosis is unclear. Here, we evaluated gene-environment interaction between multiple genetic variants including HLA genes and smoking in sarcoidosis defined by two clinical phenotypes, Löfgren’s syndrome (LS) and patients without Löfgren’s syndrome (non-LS). To quantify smoking effects in sarcoidosis, we performed a gene-environment interaction study in a Swedish population-based case-control study consisting of 3,713 individuals. Cases and controls were classified according to their cigarette smoking status and genotypes by Immunochip platform. Gene-smoking interactions were quantified by an additive interaction model using a logistic regression adjusted by sex, age and first two principal components. The estimated attributable proportion (AP) was used to quantify the interaction effect. Assessment of smoking effects with inclusion of genetic information revealed 53 (in LS) and 34 (in non-LS) SNP-smoking additive interactions at false discovery rate (FDR) below 5%. The lead signals interacting with smoking were rs12132140 (AP = 0.56, 95% CI = 0.22–0.90), *p* = 1.28e-03) in *FCRL1* for LS and rs61780312 (AP = 0.62, 95% CI = 0.28–0.90), *p* = 3e-04) in *IL23R* for non-LS. We further identified 16 genomic loci (in LS) and 13 (in non-LS) that interact with cigarette smoking. These findings suggest that sarcoidosis risk is modulated by smoking due to genetic susceptibility. Therefore, patients having certain gene variants, are at a higher risk for the disease. Consideration of individual’s genetic predisposition is crucial to quantify effects of smoking in sarcoidosis.

## Introduction

Sarcoidosis is a multi-systematic inflammatory disorder with an unknown etiology. The disease can affect any organ in the body; however, the lungs and lymphatic system are the most common targets. Sarcoidosis is best characterized by two main clinical phenotypes that describe the disease course, Löfgren’s syndrome (LS) and non-Löfgren’s syndrome (non-LS).

Prevalence and incidence of sarcoidosis varies across different age groups, ethnicities and sex^1^. In Sweden, the prevalence ranges from 152 to 215 per 100,000 and the incidence is 11.5 per 100,000 per year. Moreover, the incidence peaks in males aged 30–50 years and in females aged 50–60 years^2^.

Genetic susceptibility plays an important role in development of the disease. The major genetic factor for sarcoidosis is the HLA-DRB1*0301 allele that is located in the major histocompatibility complex (MHC) class II region. Recent evidence from genome-wide association studies highlighted that several other genetic variants outside of HLA locus generally associated with relatively small effects size on the disease susceptibility^3–7^. Particularly, a recent study by our group showed that the genetic architecture of sarcoidosis phenotypes, LS and non-LS, differed in their genetic susceptibility and activity of cells, suggesting distinct genetic architectures and molecular mechanisms between these phenotypes^8^. Moreover, in a recent study^9^ using Swedish National Patient Registry data, familial aggregation for sarcoidosis was estimated with a 3.7-fold increased disease risk. The relative risk increased (RR > 4.1) if the affected individuals had one or more relatives with the disease. Familial heritability was estimated to 39% in the Swedish population.

Epidemiological studies have reported that environmental factors also play a role in the disease development. Moreover, research findings indicate that sarcoidosis results from interplay between susceptibility genes and environmental factors^10,11^. Smoking is one of the environmental factors believed to be associated with the disease; however, evidence from epidemiological studies showed conflicting findings. For example, investigations by Ungprasert *et al*.^12^ and Carlens *et al*.^13^ reported that smokers had a lower risk of developing sarcoidosis. Similarly, Newman *et al*.^14^ observed that in the ACCESS study ever smokers had lesser odds for the disease; whereas, Visser *et al*.^15^ and Douglas *et al*.^16^ showed the disease was negatively associated with smoking. Moreover, Gupta *et al*.^17^ found no association between smoking and sarcoidosis and Janot *et al*.^18^ reported that cigarette smoking might be a risk factor for certain types of sarcoidosis. On regards to smoking effects and indices of disease, Schildge^19^ showed that smokers with sarcoidosis had a lower albumin concentration in broncoalveolar lavage fluid (BALF) compared to non-smokers^19^. Along this line, Harf at al. reported decreased levels of proteins in BALF for smokers that had sarcoidosis compared to smokers who did not have the disease and healthy controls. Clearly, in all the aforementioned studies, genetic information of smokers and non-smokers was not considered in their assessment.

In the present study, we sought to investigate the effect of smoking on sarcoidosis by taking into account genetic information in a population-based case-control study by employing a gene-environment interaction approach.

## Results

Clinical characteristics of the Swedish population-based case-control study compromising 3,713 individuals is described by defined groups ‘never smokers’ and ‘ever smokers’ as shown in Table 1. In general, 747 sarcoidosis cases (292 LS and 455 non-LS) and 2,966 healthy controls (HC) (i.e., 1,906 controls from the EIRA study and 1,060 from the EIMS study) were analyzed. Female sex was 44.5% in LS, 41.2% in non-LS and 73.8% in controls. The mean age was 38.6 years (SD = ±10.04) in LS, 42 years (SD = ±19.78) in non-LS, and 52.9 years (SD = ±18.81) in controls. Smoking distribution among never smokers and ever smokers was 46.7% and 53.3% in LS, 50.5% and 49.5% in non-LS and 45.5% and 54.5% in controls. Smoking variable distributions in LS, non-LS and HC is available in Table S0 in Supplementary Data.

**Table 1:** Clinical characteristics of the study participants consisting (n = 3,713 individuals).

	LS			Non-LS			Healthy controls	
	never smokers (n = 155)	ever smokers (n = 137)	p-value	never smokers(n = 230)	ever smokers (n = 225)	p-value	never smokers (n = 1349)	ever smokers (n = 1617)
Sex (male/female)	91/64	71/66	0.241	145/85	123/102	0.071	331/1018	446/1171
Age (mean ± sd), years	39.19 ± 8.75	36.41 ± 10.93	6.03e-07	45.50 ± 12.07	42.74 ± 12.77	5.00e-3	51.79 ± 11.87	53.89 ± 10.95
HLA-DRB1*03 positive/negative	102/53	97/40	0.381	30/187	48/162	0.011	316/1033	394/1223
**Chest X-ray stage**								
0/I/II/III/IV	1/82/33/4/1	4/61/40/2/0	—	7/45/75/35/6	4/43/65/31/7	—	—	—
**Pulmonary Function** (**mean** ± **sd**)								
VC	97.80 ± 14.42	92.38 ± 12.62	0.002	91.60 ± 15.98	86.53 ± 16.62	0.058	—	—
FVC	98.85 ± 14.32	93.78 ± 11.76	0.013	91.98 ± 16.51	87.14 ± 16.87	0.065	—	—
FEV1	96.35 ± 13.20	89.78 ± 14.79	0.001	87.29 ± 17.45	81.96 ± 18.05	0.136	—	—
TLC	94.24 ± 22.29	85.47 ± 28.25	0.001	86.46 ± 25.32	85.15 ± 22.29	0.222	—	—
DLCO	87.98 ± 21.37	82.02 ± 24.68	0.038	75.15 ± 28.95	72.53 ± 25.42	0.182	—	—
**BAL analysis** (**mean** ± **sd**)								—
% Recovery	66.28 ± 11.57	66.36 ± 12.77	0.674	64.88 ± 14.55	62.99 ± 14.78	0.118	—	—
% Viability	91.78 ± 7.79	91.42 ± 10.83	0.947	92.73 ± 4.72	90.32 ± 9.39	0.194	—	—
Total cell number (*10e6)	30.92 ± 22.98	33.17 ± 28.12	0.756	27.21 ± 15.91	34.22 ± 23.74	0.017	—	—
Cell concentration (*10e6/L)	180.50 ± 103	200.45 ± 147.61	0.432	179.66 ± 117.31	221.77 ± 163.46	0.023	—	—
**BAL differential counts** (**median ± sd**)								—
% macrophages	74 ± 13.04	76.2 ± 17.42	0.112	72.4 ± 17.04	76.90 ± 17.22	0.389	—	—
% lymphocytes	24 ± 13.22	20.6 ± 16.92	0.047	23.6 ± 16.34	21.4 ± 16.59	0.464	—	—
% neutrophils	1.2 ± 1.83	1.0 ± 2.33	0.968	0.8 ± 3.73	1.00 ± 3.48	0.632	—	—
% eosinophils	0.2 ± 0.51	0.2 ± 0.73	0.006	0.2 ± 0.83	0.2 ± 1.62	0.676	—	—
% basophils	0 ± 0.095	0 ± 0.092	0.391	0 ± 0.256	0 ± 0.245	0.165	—	—
CD4/CD8 ratio	8.25 ± 7.31	6.7 ± 7.22	0.076	4.3 ± 4.17	4.2 ± 6.15	0.86	—	—

### Smoking and risk of sarcoidosis without genetic data

Results from assessing the effect of smoking on sarcoidosis without genetic information adjusted for sex and age showed no association with the outcome. That is, LS (OR = 0.84, 95% CI = 0.64–1.09, *p* = 0.19) and non-LS (OR = 0.92, 95% CI = 0.74–1.13, *p* = 0.42).

In HLA-DRB1*03 stratified groups adjusted for sex and age, no association between smoking and disease risk was found. Results showed LS HLA-DRB1*03 positive (OR = 0.83, 95% CI = 0.56–1.23, *p* = 0.35), LS HLA-DRB1*03 negative (OR = 0.75, 95% CI = 0.48–1.16, *p* = 0.19), non-LS, HLA-DRB1*03 positive (OR = 1.46, 95% CI = 0.89–2.4, *p* = 0.14) and non-LS HLA-DRB1*03 negative (OR = 0.82, 95% CI = 0.65–1.04, *p* = 0.11).

### Gene-smoking interaction and risk of sarcoidosis

Results from additive interaction assessment in LS versus controls revealed 53 SNPs that were interacting with smoking at AP p-value < 0.05 and FDR < 0.05. The top 8 SNPs interacting with smoking at FDR < 0.01 are shown in Table 2. Complete list of results are in Table S1 in Supplementary Data. The most significant SNP-smoking interacting signal was rs12132140 (AP = 0.56, 95% CI: 0.22–0.90, *p* = 1.28e-03, FDR = 2.47e-03) located in an intron of *FCRL1* on chromosome 1. LS patients who had both risk factors, i.e., smoking and risk allele had a double exposure risk (OR = 2.92, 95% CI: 1.68–5.07) with an attributable proportion of 56% disease risk compared to LS patients who had one risk factor, i.e., smoking and no risk allele (OR = 1.18 95% CI: 0.64–2.21) or risk allele and no smoking (OR = 1.09 95% CI: 0.82–1.46).

**Table 2:** Summary of findings from gene-environment interaction between SNP and cigarette smoking in LS and non-LS, respectively at DR < 0.01 (complete list of results at FDR < 0.05 are available in Tables 1 and Table S2 in Supplementary Data.

	Marker name	CHR	Position (hg19)	Location of marker	A1/A2	CA	OR_SNP_(95% CI)	OR_SMOK_(95% CI)	OR_SNP-SMOK_(95% CI)	AP(95% CI)	AP p-val	FDR
LS	rs12132140	1	157767362	*FCRL1*	A/G	A	1.18 (0.64, 2.21)	1.09 (0.82, 1.46)	2.92 (1.68, 5.07)	0.56 (0.22, 0.90)	1.28E-03	2.47E-03
	rs115265657	1	200549451	*KIF14*	G/A	G	1.21 (0.57, 2.58)	1.12 (0.85, 1.49)	3.35 (1.7, 6.59)	0.60 (0.22, 0.98)	1.90E-03	3.67E-03
	rs62198467	2	185804581	*366* *bp 3*′ *of ZNF804A*	G/A	G	1.11 (0.66, 1.86)	1.05 (0.77, 1.42)	2.41 (1.52, 3.84)	0.52 (0.20, 0.84)	1.35E-03	2.62E-03
	rs17281154	5	66151464	*MAST4*	A/G	A	1.04 (0.61, 1.79)	1.05 (0.78, 1.42)	2.21 (1.39, 3.52)	0.50 (0.16, 0.84)	3.80E-03	7.35E-03
	rs34093877	6	106513510	*21* *kb 5*′ *of PRDM1*	G/A	G	1.70 (0.80, 3.65)	1.10 (0.83, 1.47)	4.27 (2.32, 7.89)	0.58 (0.19, 0.96)	3.38E-03	6.54E-03
	rs1060242	8	67380528	*ADHFE1*	G/A	A	1.03 (0.64, 1.67)	1.00 (0.73, 1.37)	2.06 (1.36, 3.11)	0.50 (0.18, 0.81)	1.96E-03	3.78E-03
	rs7071579	10	81910278	*4*.*6* *kb 3*′ *of ANXA11*	G/A	A	1.26 (0.81, 1.96)	1.02 (0.74, 1.41)	2.32 (1.54, 3.49)	0.45 (0.13, 0.76)	4.99E-03	9.66E-03
	rs3759321	12	6062878	*VWF*	A/G	G	1.01 (0.63, 1.61)	1.01 (0.74, 1.39)	1.96 (1.29, 2.96)	0.48 (0.16, 0.80)	3.49E-03	6.75E-03
non-LS	rs12565567	1	67651412	*IL23R*	A/G	A	1.20 (0.63, 2.28)	1.05 (0.84, 1.31)	3.05 (1.69, 5.5)	0.59 (0.24, 0.94)	8.24E-04	1.88E-03
	rs17129698	1	67654072	*IL23R*	A/G	A	1.22 (0.64, 2.32)	1.05 (0.84, 1.31)	3.05 (1.69, 5.5)	0.58 (0.23, 0.94)	1.13E-03	2.57E-03
	rs61780310	1	67659353	*IL23R*	G/A	G	1.19 (0.63, 2.27)	1.05 (0.84, 1.31)	3.03 (1.68, 5.45)	0.59 (0.24, 0.94)	8.56E-04	1.95E-03
	rs61780311	1	67661307	*IL23R*	G/C	G	1.21 (0.62, 2.37)	1.05 (0.84, 1.31)	3.24 (1.75, 5.97)	0.61 (0.27, 0.95)	4.94E-04	1.13E-03
	rs61780312	1	67661648	*IL23R*	C/A	C	1.18 (0.6, 2.31)	1.05 (0.84, 1.31)	3.23 (1.75, 5.97)	0.62 (0.28, 0.95)	3.00E-04	6.85E-04
	rs28464018	1	67662430	*IL23R*	G/A	G	1.19 (0.61, 2.32)	1.05 (0.84, 1.31)	3.23 (1.75, 5.97)	0.62 (0.28, 0.95)	3.31E-04	7.54E-04
	rs17658318	5	150322270	*ZNF300P1*	A/G	A	1.05 (0.69, 1.61)	1.01 (0.8, 1.28)	1.99 (1.32, 2.99)	0.47 (0.15, 0.78)	3.47E-03	7.92E-03
	rs10485038	6	90814274	*BACH2*	G/C	G	1.50 (0.87, 2.59)	1.05 (0.84, 1.32)	3.24 (1.87, 5.61)	0.52 (0.16, 0.88)	4.13E-03	9.42E-03
	rs57858238	6	90820248	*BACH2*	A/G	A	1.50 (0.87, 2.59)	1.05 (0.84, 1.32)	3.32 (1.91, 5.76)	0.53 (0.18, 0.88)	2.79E-03	6.37E-03
	rs10498966	6	91032094	*9*.*5* *kb 3*′ *of MIR4464*	G/A	G	1.02 (0.58, 1.81)	1.04 (0.83, 1.3)	2.36 (1.39, 4.01)	0.55 (0.21, 0.89)	1.53E-03	3.49E-03
	rs79460410	11	60777914	*CD6*	A/G	A	1.01 (0.51, 2.01)	1.06 (0.85, 1.32)	2.81 (1.47, 5.37)	0.62 (0.27, 0.97)	5.19E-04	1.19E-03
	rs75204333	16	67574408	*FAM65A*	G/A	G	1.02 (0.57, 1.82)	1.03 (0.83, 1.3)	2.30 (1.39, 3.8)	0.54 (0.20, 0.89)	2.09E-03	4.78E-03

Chr = chromosome; A1 = minor allele; A2 = major allele; CA = coded allele; MAF = minor allele frequency; OR_SNP_ = odds ratio for SNP; OR_SMOK_ = odds ratio for smoking; OR_SNP-SMOK_ = odds ratio for double exposure (SNP and smoking); AP = attributable proportion; CI = confidence interval; FDR = false discovery rate; (*) denotes SNPs in high linkage disequilibrium (LD metric R^2^ > 0.80) tagged by rs61780312.

Additive interaction analysis in non-LS versus controls highlighted 34 SNPs interacting with cigarette smoking at AP p-value < 0.05 and FDR < 0.05. The top 12 SNPs interacting with smoking at FDR < 0.01 are listed in Table 2. Complete list of results is available in Table S2 in Supplementary Data. The most significant SNP-smoking interacting signal was rs61780312 (AP = 0.62, 95% CI: 0.28–0.95, *p* = 3e-04, FDR = 6.85e-04) located in an intron of *IL23R* on chromosome 1. Non-LS patients who had both factors, i.e., risk allele and smoking had double exposure risk (OR = 3.23, 95% CI: 1.75–5.97) with an attributable proportion of 62% disease risk compared to non-LS patients with one risk factor, i.e., smoking and no risk allele (OR = 1.18, 95% CI 0.6–2.31) or risk allele and no smoking (OR = 1.05, 95% CI: 0.84–1.31).

**Figure 1:**
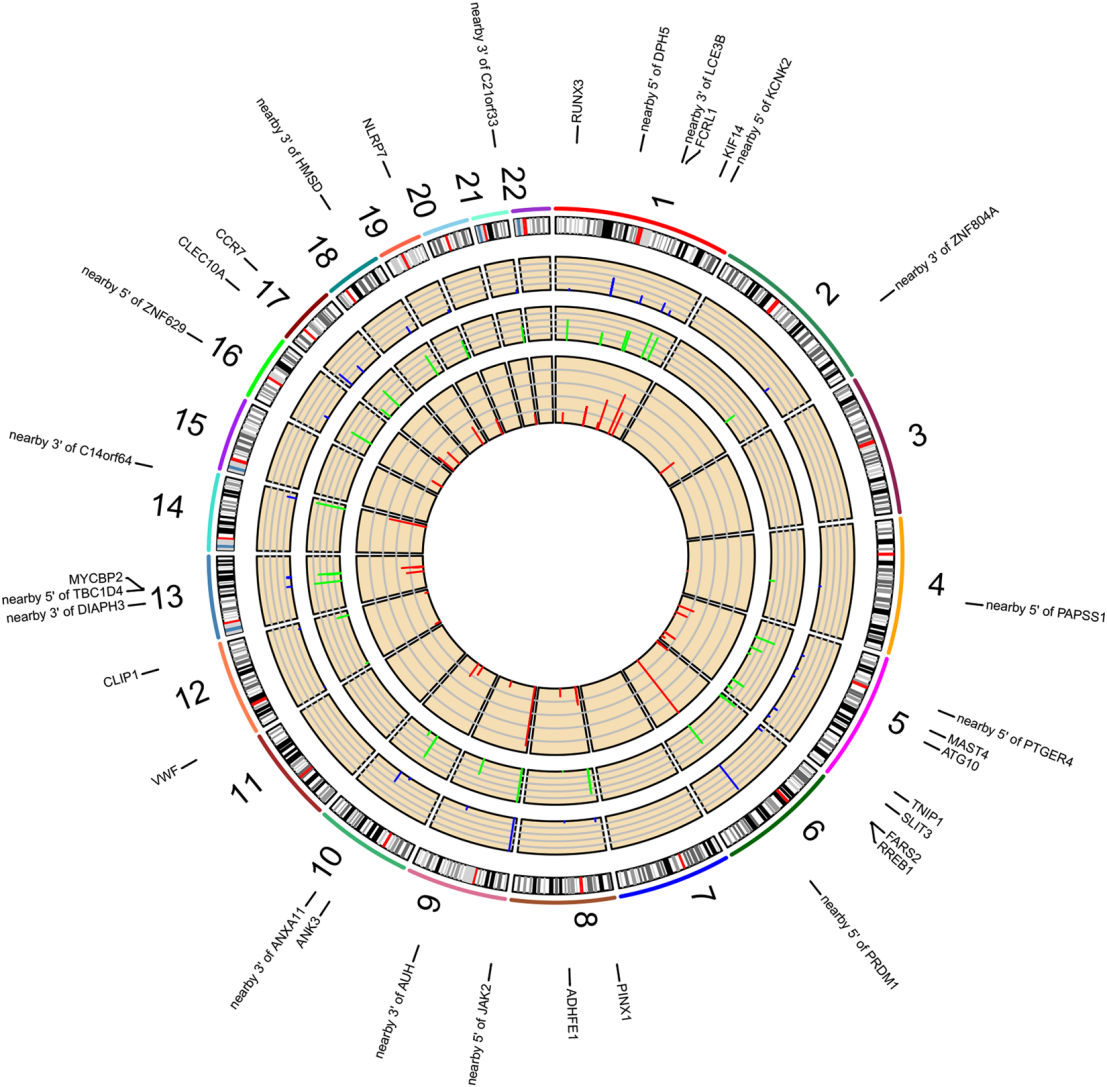
Genomic regions interacting with smoking in LS. From the center, the first circos depicts the odds ratio due to double exposure i.e., the effect of both SNP allele and smoking (in red). The second circos illustrates the effect of smoking (green). The third circos depicts the effect of the SNP allele (blue). The fourth circos shows the autosomal chromosomes (1–22) ideogram and highlights the genomic loci interacting with smoking.

**Figure 2:**
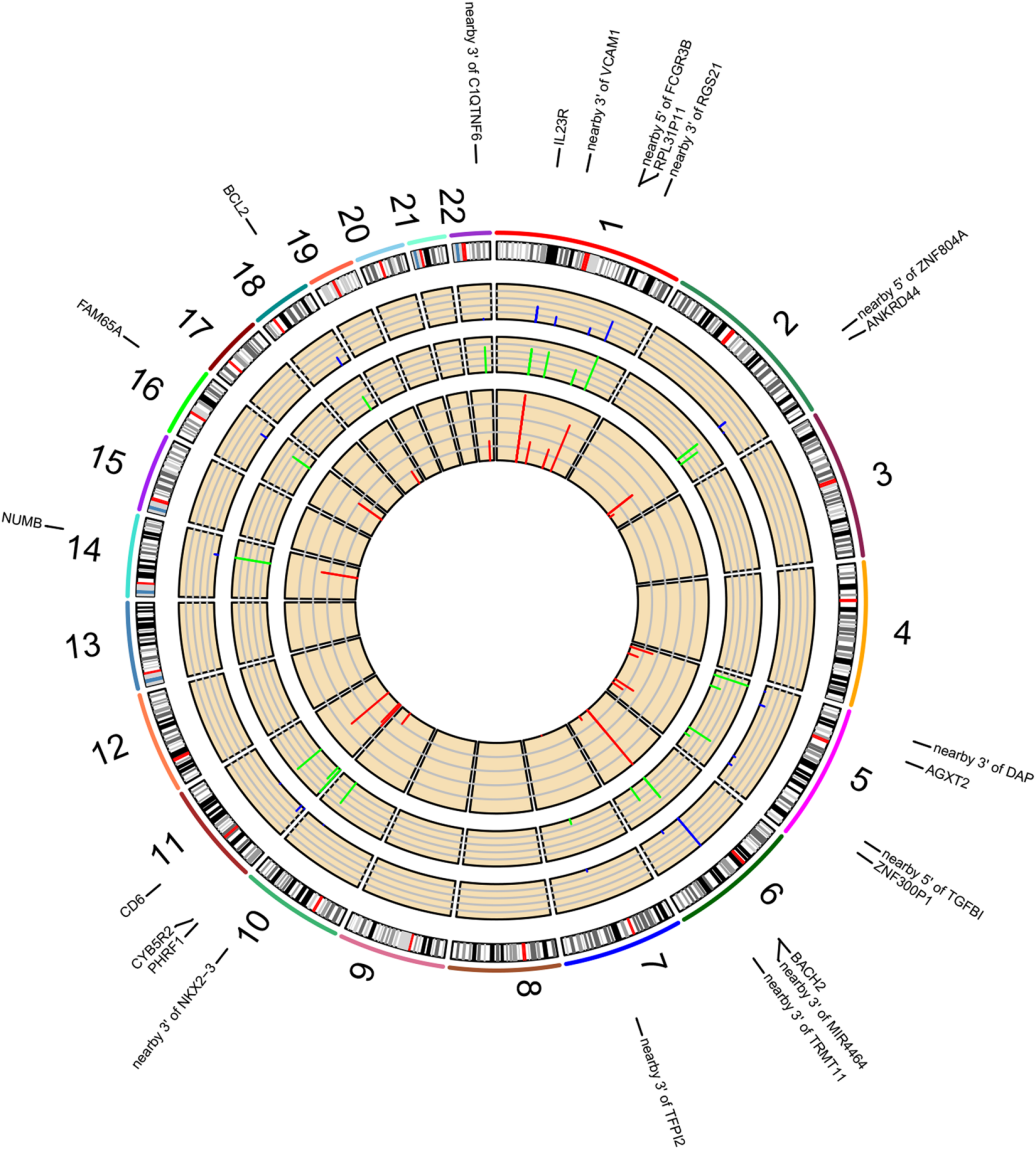
Genomic regions interacting with smoking in non-LS. From the center, the first circos depicts the odds ratio due to double exposure i.e., the effect of both SNP allele and smoking (in red). The second circos illustrates the effect of smoking (green). The third circos depicts the effect of the SNP allele (blue). The fourth circos shows the autosomal chromosomes (1–22) ideogram and highlights the genomic loci interacting with smoking.

Gene-based analysis using significant SNP-smoking interacting signals identified 16 gene regions associated with smoking in LS and 13 in non-LS (Table 3) as illustrated in Figs. 1 and 2, respectively.

**Table 3:** Summary of gene-based analysis using SNP-smoking interactions (at FDR < 0.05) located in genic regions for LS and non-LS, respectively.

	Chr	Gene	Start	Stop	Pvalue	Best-SNP	AP-pvalue
LS	1	*FCRL1*	157764192	157789940	1.33E-03	rs12132140	1.28E-03
	1	*KIF14*	200520624	200587966	2.01E-03	rs115265657	1.90E-03
	5	*MAST4*	65892175	66282351	3.88E-03	rs17281154	3.80E-03
	5	*ATG10*	81267843	81551213	2.27E-02	rs17245874	2.21E-02
	5	*TNIP1*	150409503	150444692	7.04E-03	rs3805431	6.93E-03
	5	*SLIT3*	168088737	168728133	1.09E-02	rs6890724	1.03E-02
	6	*FARS2*	5261250	5771825	5.72E-03	rs17140294	6.08E-03
	6	*RREB1*	7107829	7252213	2.05E-02	rs482393	2.03E-02
	8	*PINX1*	10622470	10697409	8.76E-03	rs17152442	8.16E-03
	8	*ADHFE1*	67344717	67381044	1.93E-03	rs1060242	1.96E-03
	12	*VWF*	6058039	6233841	3.40E-03	rs3759321	3.49E-03
	12	*CLIP1*	122755980	122907116	2.47E-02	rs11057405	2.47E-02
	13	*MYCBP2*	77618791	77901177	5.54E-03	rs34700794	5.48E-03
	17	*CLEC10A*	6977855	6983626	2.02E-02	rs11653290	2.03E-02
	17	*CCR7*	38710020	38716646	2.48E-02	rs2290065	2.49E-02
	19	*NLRP7*	55434876	55458873	5.89E-03	rs73055288	5.33E-03
non-LS	1	*IL23R*	67632168	67725662	4.59E-04	rs61780312	3.00E-04
	1	*RPL31P11*	161653484	161655042	1.09E-02	rs114384494	1.04E-02
	2	*ANKRD44*	197851385	198175521	1.79E-02	rs35272229	1.77E-02
	5	*AGXT2*	34998205	35048240	8.80E-03	rs17245714	8.93E-03
	5	*ZNF300P1*	150309997	150326146	3.14E-03	rs17658318	3.47E-03
	6	*BACH2*	90636246	91006627	2.31E-03	rs57858238	2.79E-03
	11	*CD6*	60739112	60787849	5.10E-04	rs79460410	5.19E-04
	11	*PHRF1*	576445	612222	7.32E-03	rs118042921	7.76E-03
	11	*CYB5R2*	7686325	7695474	5.13E-03	rs61729556	5.31E-03
	14	*NUMB*	73741917	73925286	6.71E-03	rs177372	6.84E-03
	16	*HSD11B2*	67465035	67471456	4.82E-03	rs5479	5.32E-03
	16	*FAM65A*	67562719	67580691	1.95E-03	rs75204333	2.09E-03
	18	*BCL2*	60790578	60986613	1.47E-02	rs4987801	1.50E-02

Results from secondary analysis where SNP-smoking interactions in LS and non-LS groups were additionally adjusted for the HLA-DRB1*03 allele revealed 106 SNP-smoking interacting signals in LS and 33 in non-LS. Comparisons from the HLA-DRB1*03 unadjusted model showed 80 additional SNP-smoking interactions in LS (Table S3 in Supplementary Data) and 3 additional SNP-smoking interactions in non-LS (Table S4 in Supplementary Data). Furthermore, stratified analysis by the HLA-DRB1*03 allele in LS and non-LS showed even higher number of SNP-interacting signals; however, due to the nonlinearity effect caused by the smaller number of samples in each strata that lead to computational limitations in the calculation of the lower and upper bound of the confidence intervals, these results are not shown (Data available upon request).

### Network analysis on SNP-smoking interactions

Results from evaluating LS SNP-smoking interactions using network analysis identified a top gene network marked by *MAST4*, *TNIP1*, *SLIT3*, and *RUNX3* that highlighted five GO processes (Table S5 in Supplementary Data). The most significant GO process was positive regulation of nitrogen compound metabolic process (87.2%; *p* = 6.364e-25). Likewise, network analysis based on non-LS SNP-smoking interactions revealed a top gene network marked by *BACH2*, *NUMB*, *IL23R*, and *CD6* that pinpointed five key GO processes (Table S5 in Supplementary Data). The most significant GO process was positive regulation of cellular process (91.7%; *p* = 9.839e-19).

## Discussion

Cigarette smoking is a harmful environmental factor that poses deleterious effects in the human body and is certainly the foremost cause of morbidity and mortality in smoking-related diseases in industrialized countries. It is worth noting that in addition to nicotine that is a fast-acting additive substance, there are other several compounds and carcinogens found in tobacco, including hydrogen cyanide, formaldehyde, lead, arsenic, ammonia, radioactive elements (e.g. uranium), benzene, carbon monoxide, nitrosamines, and polyclinic aromatic hydrocarbons^20,21^ that are toxic to human health.

The present work is by far the largest study conducted for investigating the effect of cigarette smoking and risk of sarcoidosis by considering genetic information of smokers and non-smokers. Our findings suggests that for some patients having certain gene variants, smoking modulates their disease risk.

In addition to the recently discovered genetic susceptibility of sarcoidosis clinical phenotypes, LS and non-LS^8^, this study provides new information regarding genomic regions that interac with cigarette smoking as quantified by the attributable proportion (AP). The results of our study showed that smoking modulates disease risk by 56% in LS and 62% in non-LS if patients have both risk factors; that is, the risk allele and smoking, and henceforth offering new insights on the disease susceptibility and its interaction to smoking exposure.

SNP-smoking interaction analysis revealed 53 genetic variants interacting with cigarette smoking in LS, highlighting lead signal rs12132140 located in an intron of *FCRL1* (Entrez Gene: 115350) with an increased disease risk of 56% defined by the estimated AP. The *FCRL1* (Fc receptor-like 1) gene encodes a membrane protein of the immunoglobulin superfamily receptor that is preferentially expressed by B cells. B-cell infiltrates have been observed in granulomatous tissues^22,23^ and Fc receptor-like homologs have been reported to be downregulated in smokers and ex-smokers^24^. Moreover, *FCRL1* has been found to be associated with smoking cessation^25^ and several autoimmune diseases^26,27^. Thus, it is plausible to consider a functional mechanism between *FCRL1* and cigarette smoking in sarcoidosis. Furthermore, additional interacting signals located in intronic regions of *KIF14*, *MAST4* and *VWF* and nearby *ZNF804A*, *PRDM1*, and *ANXA11* also showed increased disease risk by the estimated AP. These findings warrant further investigation as such loci may play a role in inflammatory signaling pathways when interacting with smoking in sarcoidosis and perhaps in other immune-mediated diseases^28^.

In non-LS, SNP-smoking interactions revealed 34 genetic variants interacting with smoking. The top signals were located in introns of *IL23R* (Entrez Gene: 149233) that were in high linkage disequilibrium (LD, *r*^*2*^ > 0.8) and tagged by rs12565567. The most significant SNP-smoking interacting signal was rs61780312 (in LD with rs12565567, *r*^*2*^ = 0.84) that showed a 62% increased disease risk. Interestingly, *IL23R* is a known risk locus for sarcoidosis^29,30^, smoking behavior^25,31^, and autoimmune diseases, i.e., Crohn’ disease^31^, ulcerative colitis^32^, psoriasis^33,34^, and ankylosing spondylitis^35^. The protein encoded by the *IL23R* (Interleukin 23 receptor) gene is pro-inflammatory cytokine that stimulates T-helper 17 (Th17) cells and other cytokines in cell-mediated inflammatory processes^36^. Th17-cells have been suggested to play a role in the pathogenesis of sarcoidosis^37,38^, primarily in granuloma formation^37,39^. Recently, newly identified Th17.1-cells in sarcoidosis and IL-17A, which has a specific role in the lungs, prompt new questions for their functional role as may be implicated in separate immune molecular mechanisms in LS and non-LS sarcoidosis^40,41^. Additional significant SNP-smoking interacting signals located in intronic regions of *ZNF300P1*, *BACH2*, *CD6* and *FAM65A*, and nearby *mir4464* were also observed, as shown in Table 3. These genomic loci warrant further examination as such loci have been reported to be associated with various autoimmune diseases, suggesting a plausible role in autoimmunity^28^ and which may interact with smoking.

It is worth highlighting that without genetic information, the relationship between smoking and risk of sarcoidosis showed no association which concurs with previous findings^17,42^. Nevertheless, when the genetic information of the individuals was considered to evaluate the relationship between smoking and disease risk, this assessment enabled us to identify genomic regions interacting with smoking that showed an increased disease risk. Such increased effect may be explained by the underlying synergy between genetic susceptibility of sarcoidosis and smoking. Additionally, SNP-smoking interactions adjusted for the HLA-DRB1*03 allele, which is a marker of good disease prognosis particularly in LS patients, showed additional SNP-smoking interacting regions, suggesting that conditioning for *HLA-DRB1* facilitates to capture additional loci that interact with cigarette smoking and that are unbiased by the effect of *HLA-DRB1*. Notably, *HLA-DRB1* variations found to be interacting with cigarette smoking, often modulate and/or increase disease risk in autoimmune diseases, such as in rheumatoid arthritis^43^ and multiple sclerosis^44^. Henceforth, the functional role of the *HLA-DRB1* and smoking in sarcoidosis shall be further explored.

Interestingly, results from network analysis pinpointed significant gene networks highlighting GO processes, such as nitrogen compound metabolic process and response to organic substances, which may be triggered by cigarette smoking and requires therefore further investigation.

Considerable evidence in the literature suggest that cigarette smoking is a major risk factor for increased risk of disease in cardiovascular, respiratory, cancer and multiple inflammatory chronic diseases^45,46^. Yet, there are exceptions in where smoking seems to have an inverse relationship with disease, such as in ulcerative colitis (UC)^47–49^ and hypersensitivy pneumonitis (HP)^50,51^. Within UC and HP, cigarette smoking is suggestive to have a favorable effect on the disease. However, the postulated protective effect lacks a clear long-term benefit as its duration is only temporary and has been reported to worsen the prognosis of the disease^52^. In contrast, the effect of smoking in other autoimmune diseases, such in rheumatoid arthritis (RA) is more evident, where the exposure of cigarette smoking increases disease risk and has been demonstrated to interact with various risk genes including *HLA-DRB1*^43^. Such observed gene-smoking interactions may be explained by smoking causing peptidyl arginine deiminase (PAD) activation which then triggers citrullination in the lungs along with other compounds found in tobacco that activate antigen presenting cells (APCs) in the pulmonary compartment^53,54^. In the case of sarcoidosis, the risk effect of cigarette smoking has not been quite clear as various studies reported conflicting findings. For instance, Valeyre *et al*. showed that although smokers with sarcoidosis had lower CD4:CD8 ratio, smoking increased indices of disease activity, such as serum angiotensin converting enzyme (srACE) and pulmonary gallium-67^55^. Whereas Julian *et al*.^56^ showed an improved responsiveness of toll-like receptors (TLR2 and TLR9) in active sarcoidosis observed in blood using nicotine treatment. Toll-like receptors (TLRs) tend to be highly expressed upon stimulation of the microbiota and have the ability to trigger proinflammatory responses^57,58^; however, the implication of TLRs in the pathogenesis of sarcoidosis is yet to be elucidated. Perhaps one way to understand the role of TLRs and smoking in sarcoidosis would be to examine the lung microbiome among smokers and non-smokers, which may shed the light on the functional mechanisms triggered by nicotine exposure.

In the context of effect measurement of cigarette smoking, it is important to stress that although nicotine substance (e.g., transdermal nicotine patches) is the preferred model for measuring smoking effects, nicotine is not the only compound in tobacco. A cigarette has about 600 ingredients, which when burned it creates more than 7,000 chemicals. Therefore, there are other chemicals in addition to nicotine which have the potential to pose a health hazard and be the cause of increased and/or modulated disease risk. Swedish moist snuff, for example, which gives a very high concentration of nicotine lasting for long hours was shown to have no effect on the risk of sarcoidosis, suggesting that inhaled non-nicotine compounds are more likely to influence disease risk^13^. Notably, the quantitative effect of nicotine on autoimmunity is a complex measure and therefore its effect on the immune system requires meticulous examinations^59^. Certainly, more comprehensive studies to compare nicotine efficiency and safety effects in patients with autoimmune diseases, as well as documented adverse effects of current nicotine products are needed. As multifactorial diseases often result from the combination of gene-gene and gene-environment interactions, it is crucial that both genetic and environmental factors are assessed together and not independently. Given the sarcoidosis is a complex inflammatory disease with a strong genetic predisposition, it is worth considering the genetic data of individuals affected by the disease along with detailed information on environmental exposures, such as cigarette smoking by means of epistasis assessment.

In summary, by employing an approach such as gene-smoking interaction as presented in this study, we were able us to quantify the effects of smoking interacting with genetic variants of patients with sarcoidosis. Our work highlights the significance of assessing the effect of smoking on sarcoidosis by means of gene-environment interaction analysis which pinpointed various genomic loci that require validation in other cohorts and warrant further investigation including future functional studies that can elucidate on the molecular mechanisms responsible for the observed gene-smoking interactions.

### Limitations of the study

While a substantial number of SNPs showing statistically significant interaction with cigarette smoking were identified even after adjustment for multiple testing, it is worth mentioning that there are some limitations that ought to be noted. First, smoking habits in cases and controls were assessed by two different questionnaires. Since there were different definitions for current and past smokers in the questionnaires of the controls (EIRA and EIMS studies) and cases, there is a possibility for a discrepancy that could produce some bias on the exposure variable. In order to address this issue, we categorized the smoking variable into “never-smokers” and “ever-smokers”. The latter category included current and past smokers. Second, differences in sample size among male and female groups in cases and controls shall be noted. The percentage of males among controls was around 27% (777) compared to 73% of females. In order to address sex disproportionality, we included sex as a covariate in all analyses conducted. Notably, other confounders, such as occupational exposure could impact the factor related to the exposure and the outcome. However, these variables are not available for our study. Despite these limitations, the study has a major strength – instead of focusing solely on the assessment of association between cigarette smoking and sarcoidosis, it evaluates the gene-environment interaction between sarcoidosis-associated variants (in both LS and non-LS phenotypes) and cigarette smoking. Considering that sarcoidosis has a strong genetic predisposition^8^, it is important to integrate genetic information while assessing relationship between disease and environmental exposures. Furthermore, this study distinguishes between different sarcoidosis phenotypes (LS and non-LS), giving a major advantage for understanding the complexity of the disease. Clear differences in the genetic susceptibility of LS and non-LS point at different biological mechanisms that drive pathogenesis of sarcoidosis.

## Methods

### Participants and study design

A population-based case-control study consisting of 3,713 individuals was designed. Specifically, 747 sarcoidosis cases (292 LS and 455 non-LS) and 2,966 healthy controls (1,906 controls from the Epidemiological Investigation of Rheumatoid Arthritis (EIRA) study and 1,060 controls from the Epidemiological Investigation of Multiple Sclerosis (EIMS) study) with smoking data were included. All included individuals had a self-reported white European ancestry. All studies had protocols approved by the Regional Ethical Board in Stockholm, Sweden. Participants included in the study provided written informed consent and gave permission to use their DNA for research purposes. All methods were carried out accordance with relevant guidelines and regulations.

Briefly, sarcoidosis cases were selected from a local patient registry, which was established in year 2007 at the Respiratory Division, Department of Medicine, at Karolinska Institutet University Hospital Solna, Sweden. Patients registered in the sarcoidosis local registry are residents from different areas in Sweden. The diagnosis of sarcoidosis in the case group was based on clinical symptoms and radiographic indications, as well as elevated CD4/CD8 ratio from broncoalveolar lavage fluid and positive biopsies for sarcoid granulomas^60^.

Granulomas were defined as sarcoid, when other causes were excluded according to the criteria proposed by WASOG^61^. The patient was diagnosed with Löfgren’s syndrome, if patient had an acute beginning of the disease, which included fever, bilateral hilar lymphadenopathy, bilateral arthritis of ankles and (or) erythema nodosum^60^. Blood from patients suffering from sarcoidosis was collected during diagnostic routine at the Department of Medicine, Respiratory Division at Karolinska Institutet University Hospital in Solna, Sweden.

Individuals in the control group (n = 2,966) did not have sarcoidosis diagnosis and were extracted from the EIRA study (n = 1,906) and EIMS study (n = 1,060). Further details on the EIRA and EIMS study are available elsewhere^62,63^.

### Smoking data

In the control group, smoking information from individuals in the EIRA and EIMS studies was obtained from questionnaires as described previously^63,64^. Briefly, questions on smoking included current and past smoking status, years and frequency of smoking. Control subjects who reported smoking during the index year were classified as current smokers, while subjects who quit smoking the year before the index year were defined as ex-smokers. Individuals who smoked occasionally were defined as non-regular smokers. Individuals who answered that never smoked in the index year or never smoked in their life were categorized as non-smokers. Thus, smoking categories consisted of current smokers, ex-smokers and non-smokers.

In the sarcoidosis group, smoking information was obtained by questionnaire. For new patients questionnaire was filled in at the first visit after patient’s bronchoscopy procedure. For older patients who had the disease diagnosis several years ago before the survey was established, they were given the questionnaire when possible. The questionnaire included questions about the smoking status, category of smoking (i.e., cigarettes, cigars, pipe, and moist snug), number of cigarettes per day, and smoking history. Number of pack-years was not recorded. Based on their smoking habits, participants were then classified as current smokers, ex-smokers and never smokers. Current and ex-smokers were further classified as regular and non-regular smokers. For this study, the smoking variable was defined in two categories: (1) “ever-smokers” that included ex-smokers, current smokers, and non-regular smokers and (2) “never-smokers” that included subjects who never smoked. A summary of the smoking variable distribution in LS, non-LS and healthy controls (HC) is available in Table S0 in Supplementary Data.

### Genotyping for cases and controls

The blood of all cases was previously collected at the time of diagnosis of the disease and subsequently DNA was extracted and genotyped for 186 K SNPs using the Illumina Immunochip version 1 platform. The genotyped data of cases is described in the study by Rivera *et al*.^8^. Similarly, the blood of all healthy controls was collected by the dedicated research nurses immediately next to the time of receiving response to questionnaire. DNA of controls was also genotyped on the Illumina Immunochip version 1 and is further described elsewhere^65,66^. In this study, 1,058 controls from EIMS, 1,905 controls from EIRA and 731 cases (290 LS and 441 non-LS) that passed genotyping quality control filtering were analyzed throughout the study.

### Statistical Methods

Population descriptive analysis was performed using IBM SPSS Statistics for Windows, version 24.0. (Armonk, NY, USA) software. Proportions defined by percentages were calculated to show distributions between sex (and age) and smoking habits in controls and cases (LS and non-LS). Clinical variables including chest X-ray, pulmonary function, broncoalveolar lavage (BAL) analysis, and HLA information among ‘never smokers’ and ‘ever smokers’ in LS and non-LS groups were analyzed using the Mann-Whitney U test.

Association test of smoking in sarcoidosis was tested in LS and non-LS, respectively using a multivariable logistic regression model. The binary outcome (dependent variable) was regressed on smoking adjusted by sex and age. Odds ratios (OR) with 95% confidence intervals (CI) and p-values were calculated.

Gene-Environment interaction analysis was performed in LS and non-LS groups compared to healthy controls using a logistic regression model and in-house designed method for gene-environment interaction analysis, as implemented in GEISA software^67^, an updated version of the original GEIRA software^68^. The additive interaction was based on a dominant model and consisted of the binary outcome (LS versus controls or non-LS versus controls) as dependent variable regressed on independent variables: SNP, smoking, and interaction term (SNP and smoking) and adjusted for age, sex and first two principal components (e.g., PC1 and PC2) as covariates. Principal components were calculated using PCA analysis that was performed using pruned genotype data (38,027 SNPs) and EIGENSOFT software^69^. Genotype pruning was calculated using SNP LD-based function (and *r*^*2*^ = 0.25) as implemented in PLINK v.1.07 sofware^70^. To quantify gene-environment interaction between genetic variant and smoking, the attributable proportion (AP) was considered as a quantification metric. Further details on the calculation of the effect of the interaction defined by the AP is available in^71^. Briefly, the AP was calculated based on combinations of the risks derived from the interactions among the assessment of the two risk factors, i.e., SNP and smoking. Combination groups were coded as 11 = SNP and smoking present, 01 = smoking present and SNP absent, 10 = SNP present and smoking absent, and 00 = SNP and smoking absent. In mathematical terms, the AP was defined as (OR_11_ − OR_10_ − OR_10_ + OR_00_)/OR_11_, where OR_11_ was the odds ratio of having both risk factors (SNP and smoking), OR_01_ and OR_10_ were the odds ratios of having one risk factor either SNP or smoking, and OR_00_ was the odds ratio of having no risk factors (in the GEISA software, OR_00_ = 1). The range of the AP is defined between −1 and 1, where 0 indicated no interaction between the two risk factors. The 95% confidence intervals of AP and p-value were calculated, as well as the odds ratios (ORs) and 95% confidence intervals for the different combinations groups. Additionally, to correct for multiple testing, the false discovery rate (FDR) was calculated using the AP p-values and the p.adjust function in R package^72^. In this study, we focused on identifying SNP-smoking interaction signals that only increase disease risk, thus a significant SNP-smoking interaction signal was considered when AP > 0, AP p-value < 0.05 and FDR < 0.05 were observed.

In a secondary analyses, we adjusted the SNP-smoking interaction signals in LS and non-LS for the effect of the HLA-DRB1*03 allele in addition to previous defined covariates (age, sex, PC1 and PC2). *HLA-DRB1* alleles were typed in sarcoidosis cases and imputed in the controls. HLA-imputation was performed using SNP2HLA software^73^. Furthermore, if more than one significant SNP interacting with smoking localized on the same locus, tag SNPs were determined using information of linkage disequilibrium among the SNPs located in the same gene and AP p-values as selection criteria, as implemented in PriorityPruner v.0.1.4 software (http://prioritypruner.sourceforge.net).

Gene-based analysis was performed based on significant SNPs interacting with smoking that localized in genic regions (e.g., 5′ or 3′ untranslated regions, exons, and introns) using the VErsatile Gene-based Association Study version 2 (VEGAS2) software^74^. Gene-based association significance was computed by performing 10^6^ permutations, which suffices to account for multiple testing correction of around 25,000 tests.

Network analysis was implemented using a state-of-the-art network algorithm as implemented in MetaCore™ (Thomson Reuters, New York, NY, USA) integrated software. The algorithm is a variant of the shortest path with main parameters of 1) relative enrichment with the uploaded data, and 2) relative saturation of networks with canonical pathways. The networks are prioritized based on the number of fragments of canonical pathways on the network. The algorithm uses the set of SNPs interacting with smoking as seed “nodes” and interactions from MetaCore™/MetaDrug™ database as “edges”.

## Supplementary information

Supplementary Info
