# Supplementary material for: A Gene–Environment Interaction Between Smoking and Gene polymorphisms Provides a High Risk of Two Subgroups of Sarcoidosis
Source: Sci Rep. 2019 Dec 9;9:18633. doi: 10.1038/s41598-019-54612-1 (PMC6901455; doi:10.1038/s41598-019-54612-1)
Supplement: Supplementary file 1 — Supplementary Info [file 41598_2019_54612_MOESM1_ESM.pdf]

# Supplementary Data

**A Gene–Environment Interaction Between Smoking and Gene polymorphisms Provides a High Risk of Two Subgroups of Sarcoidosis**

Natalia V Rivera[1,2*], Karina Patasova[1], Susanna Kullberg[1], Lina Marcela Diaz-Gallo[2], Tomoko Iseda[1], Camilla Bengtsson[3], Lars Alfredsson[3], Anders Eklund[1], Ingrid Kockum[4], Johan Grunewald[1+], and Leonid Padyukov[2+]

[1]Division of Respiratory Medicine, Department of Medicine Solna, Karolinska Institutet, Karolinska University Hospital, SE-171 76 Stockholm, Sweden

[2]Division of Rheumatology, Department of Medicine Solna, Karolinska Institutet, Karolinska University Hospital, SE-171 76 Stockholm, Sweden

[3]Institute of Environmental Medicine (IMM), Karolinska Institutet, SE-171 77 Stockholm, Sweden

[4]Neuroimmunology Unit, Department of Clinical Neuroscience, Karolinska Institutet, SE-171 76 Stockholm, Sweden.

## SUPPLEMENTARY FIGURES

**FIGURE S1.** Genomic regions interacting with smoking in LS after adjusting for *HLA-DRB1*03*

**FIGURE S2.** Genomic regions interacting with smoking in non-LS after adjusting for *HLA-DRB1*03*

## SUPPLEMENTARY TABLES

**TABLE S0.** Summary of smoking variable distribution in LS, non-LS and healthy controls (HC)

**TABLE S1.** Summary of findings from gene-environment interaction between cigarette smoking in LS at FDR < 0.05

**TABLE S2.** Summary of findings from gene-environment interaction between cigarette smoking in non-LS at FDR < 0.05

**TABLE S3.** Summary of findings from gene-environment interaction between cigarette smoking in LS after adding adjustment for *HLA-DRB1*03* at FDR < 0.05

**TABLE S4.** Summary of findings from gene-environment interaction between cigarette smoking in non-LS after adding adjustment for *HLA-DRB1*03* at FDR < 0.05

**TABLE S5.** Summary of significant gene networks based on SNP-smoking interactions in LS and non-LS, respectively at FDR < 0.05

**FIGURE S1.** Genomic regions interacting with smoking in LS after adjusting for *HLA-DRB1*03*

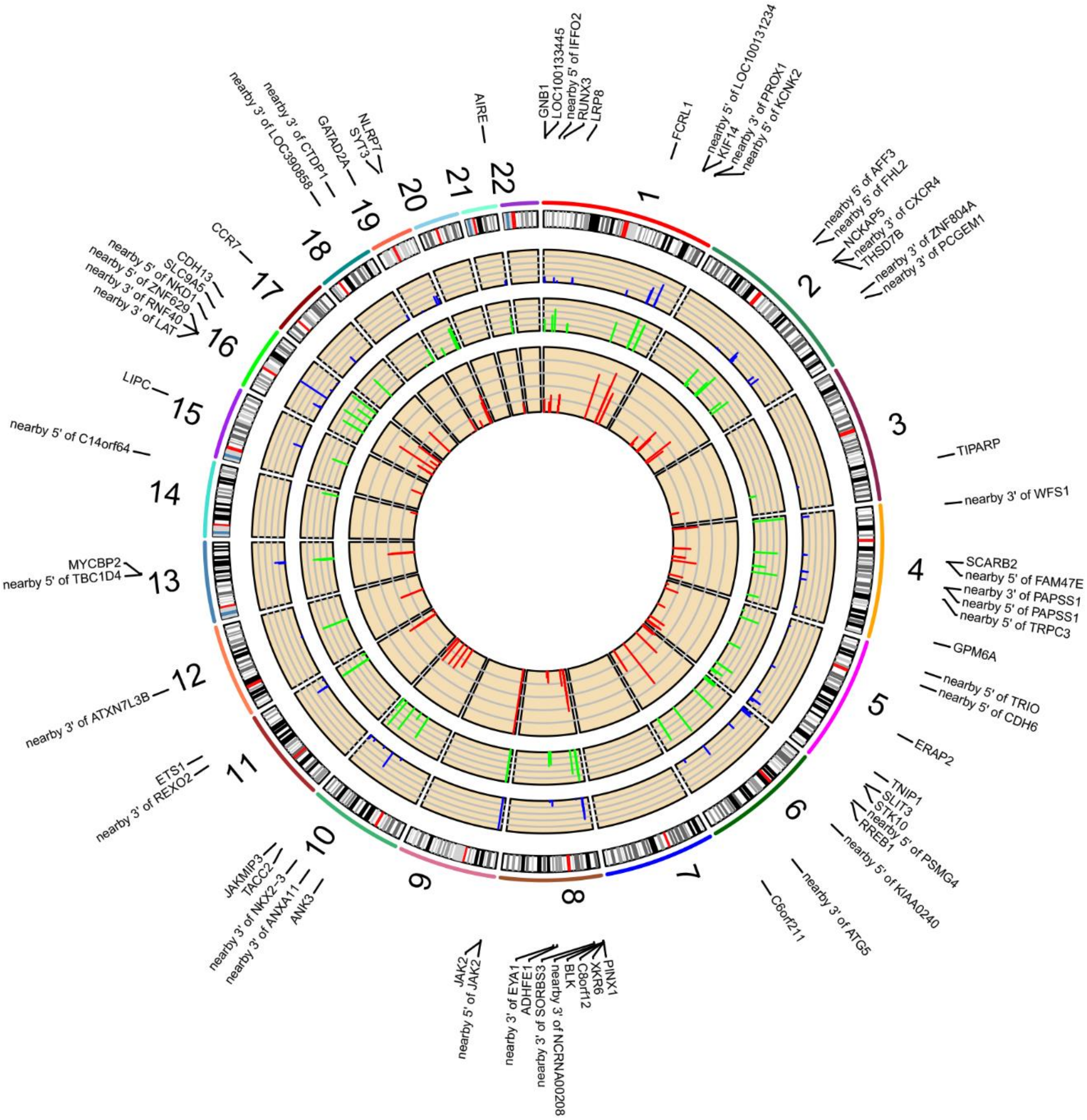


From the center, the first circos depicts the odds ratio due to double exposure i.e., the effect of risk allele and smoking shown in red color. The second circos illustrate the odds ratio due to environmental factor (smoking) shown in green color. The third circos depicts the odds ratio due to the risk allele (SNP) shown in blue color. The fourth circos shows the chromosomes (autosomal 1-22) ideogram and highlights the genomic loci interacting with smoking.

**FIGURE S2.** Genomic regions interacting with smoking in non-LS after adjusting for *HLA-DRB1*03*

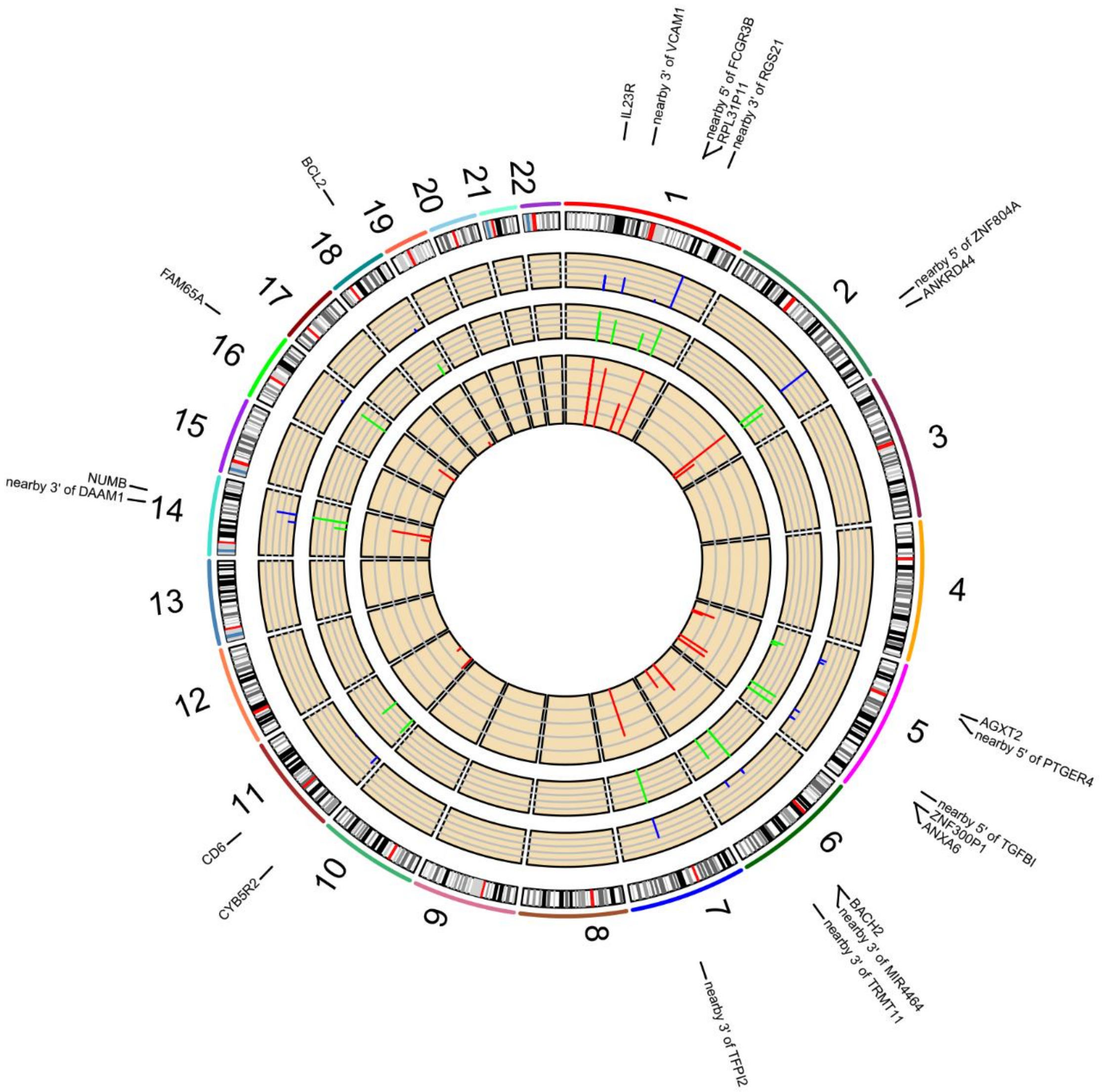


From the center, the first circos depicts the odds ratio due to double exposure i.e., the effect of risk allele and smoking shown in red color. The second circos illustrate the odds ratio due to environmental factor (smoking) shown in green color. The third circos depicts the odds ratio due to the risk allele (SNP) shown in blue color. The fourth circos shows the chromosomes (autosomal 1-22) ideogram and highlights the genomic loci interacting with smoking.

**TABLE S0. Summary of smoking variable distribution in LS, non-LS and healthy controls (HC)**

| Smoking variable | LS (n = 290) | non-LS (n = 441) | HC (n = 3163) | Total |
|---|---|---|---|---|
| ex smoker | 76 | 141 | 826 | 1043 |
| current smoker | 50 | 72 | 633 | 755 |
| non-regular smoker | 10 | 6 | 155 | 171 |
| never smoker | 154 | 222 | 1349 | 1725 |

| Dichotamized smoking variable | LS (n = 290) | non-LS (n = 441) | HC (n = 3163) | Total |
|---|---|---|---|---|
| ever smoker | 136 | 219 | 1614 | 1969 |
| never smoker | 154 | 222 | 1349 | 1725 |

**TABLE S1.** Summary of findings from gene-environment interaction between cigarette smoking in LS at FDR < 0.05

| Marker name | CHR | Position (hg19) | Marker location w RefSeq Gene | Minor/Major alleles | coded allele | $OR_{SNP\text{-}SMOK}$ | 95%CI | $OR_{SNP}$ | 95%CI | $OR_{SMOK}$ | 95%CI | AP | 95%CI | AP p-val | FDR | Best tag SNP | LD $r^2$ |
|---|---|---|---|---|---|---|---|---|---|---|---|---|---|---|---|---|---|
| rs12117581 | 1 | 25272809 | *RUNX3* | A/G | A | 2.13 | (1.28, 3.55) | 1.03 | (0.58, 1.83) | 1.09 | (0.81, 1.46) | 0.48 | (0.09, 0.86) | 1.46E-02 | 2.82E-02 | rs12117581 | 1 |
| rs35576543 | 1 | 101532242 | *41kb 5' of DPH5* | G/A | G | 2.48 | (1.61, 3.82) | 1.39 | (0.85, 2.29) | 1.05 | (0.77, 1.43) | 0.42 | (0.06, 0.77) | 2.03E-02 | 3.92E-02 | rs35576543 | 1 |
| rs17409743 | 1 | 101540050 | *49kb 5' of DPH5* | G/A | G | 2.48 | (1.61, 3.82) | 1.40 | (0.85, 2.3) | 1.05 | (0.77, 1.43) | 0.42 | (0.06, 0.77) | 2.16E-02 | 4.16E-02 | rs35576543 | 1 |
| rs10493943 | 1 | 101541367 | *50kb 5' of DPH5* | G/A | G | 2.48 | (1.61, 3.82) | 1.40 | (0.85, 2.31) | 1.05 | (0.77, 1.43) | 0.41 | (0.06, 0.77) | 2.27E-02 | 4.38E-02 | rs35576543 | 1 |
| rs4409674 | 1 | 101543389 | *52kb 5' of DPH5* | A/C | A | 2.48 | (1.61, 3.82) | 1.39 | (0.85, 2.29) | 1.05 | (0.77, 1.43) | 0.42 | (0.06, 0.77) | 2.03E-02 | 3.92E-02 | rs35576543 | 1 |
| rs12741164 | 1 | 101551513 | *60kb 5' of DPH5* | A/C | A | 2.48 | (1.61, 3.82) | 1.40 | (0.85, 2.31) | 1.05 | (0.77, 1.43) | 0.42 | (0.06, 0.77) | 2.23E-02 | 4.30E-02 | rs35576543 | 1 |
| rs35918661 | 1 | 101564836 | *73kb 5' of DPH5* | A/G | A | 2.47 | (1.61, 3.8) | 1.39 | (0.85, 2.29) | 1.05 | (0.77, 1.43) | 0.42 | (0.06, 0.77) | 2.19E-02 | 4.22E-02 | rs35576543 | 0.998 |
| rs7533697 | 1 | 101565235 | *74kb 5' of DPH5* | A/G | A | 2.47 | (1.61, 3.8) | 1.39 | (0.85, 2.29) | 1.05 | (0.77, 1.43) | 0.42 | (0.06, 0.77) | 2.19E-02 | 4.22E-02 | rs35576543 | 0.998 |
| rs12746449 | 1 | 101589082 | *98kb 5' of DPH5* | A/G | A | 2.33 | (1.53, 3.55) | 1.33 | (0.81, 2.17) | 1.04 | (0.76, 1.43) | 0.41 | (0.05, 0.77) | 2.38E-02 | 4.58E-02 | rs35576543 | 0.882 |
| rs7529431 | 1 | 101599239 | *103kb 5' of S1PR1* | C/G | C | 2.23 | (1.45, 3.44) | 1.23 | (0.74, 2.06) | 1.05 | (0.77, 1.43) | 0.43 | (0.06, 0.79) | 2.11E-02 | 4.07E-02 | rs35576543 | 0.827 |
| rs36106538 | 1 | 101601162 | *101kb 5' of S1PR1* | G/A | G | 2.24 | (1.45, 3.46) | 1.24 | (0.74, 2.06) | 1.05 | (0.77, 1.43) | 0.43 | (0.06, 0.79) | 2.10E-02 | 4.05E-02 | rs35576543 | 0.828 |
| rs7538646 | 1 | 101601911 | *100kb 5' of S1PR1* | A/G | A | 2.24 | (1.45, 3.45) | 1.24 | (0.74, 2.06) | 1.05 | (0.77, 1.43) | 0.43 | (0.06, 0.79) | 2.12E-02 | 4.09E-02 | rs35576543 | 0.829 |
| rs17450565 | 1 | 101601930 | *100kb 5' of S1PR1* | G/A | G | 2.24 | (1.45, 3.46) | 1.25 | (0.75, 2.09) | 1.05 | (0.77, 1.43) | 0.42 | (0.05, 0.78) | 2.49E-02 | 4.80E-02 | rs35576543 | 0.828 |
| rs12034595 | 1 | 152590565 | *4kb 3' of LCE3B* | A/C | A | 2.01 | (1.23, 3.29) | 1.02 | (0.59, 1.78) | 1.09 | (0.81, 1.47) | 0.45 | (0.06, 0.84) | 2.45E-02 | 4.71E-02 | rs12034595 | 1 |
| rs12132140 | 1 | 157767362 | *FCRL1* | A/G | A | 2.92 | (1.68, 5.07) | 1.18 | (0.64, 2.21) | 1.09 | (0.82, 1.46) | 0.56 | (0.22, 0.9) | 1.28E-03 | 2.47E-03 | rs12132140 | 1 |
| rs115265657 | 1 | 200549451 | *KIF14* | G/A | G | 3.35 | (1.7, 6.59) | 1.21 | (0.57, 2.58) | 1.12 | (0.85, 1.49) | 0.60 | (0.22, 0.98) | 1.90E-03 | 3.67E-03 | rs115265657 | 1 |
| rs12083512 | 1 | 215057759 | *121kb 5' of KCNK2* | G/A | G | 2.68 | (1.46, 4.9) | 1.10 | (0.57, 2.14) | 1.11 | (0.84, 1.48) | 0.55 | (0.16, 0.93) | 5.21E-03 | 1.01E-02 | rs12083512 | 1 |
| rs62198467 | 2 | 185804581 | *366bp 3' of ZNF804A* | G/A | G | 2.41 | (1.52, 3.84) | 1.11 | (0.66, 1.86) | 1.05 | (0.77, 1.42) | 0.52 | (0.2, 0.84) | 1.35E-03 | 2.62E-03 | rs62198467 | 1 |
| rs7665274 | 4 | 108690915 | *49kb 5' of PAPSS1* | G/A | G | 1.77 | (1.19, 2.64) | 1.03 | (0.66, 1.61) | 1.02 | (0.74, 1.41) | 0.41 | (0.06, 0.75) | 2.20E-02 | 4.23E-02 | rs7665274 | 1 |
| rs62358228 | 5 | 40295128 | *385kb 5' of PTGER4* | G/A | G | 2.36 | (1.38, 4.06) | 1.05 | (0.56, 1.96) | 1.09 | (0.82, 1.46) | 0.52 | (0.14, 0.9) | 7.08E-03 | 1.37E-02 | rs62358228 | 1 |
| rs17281154 | 5 | 66151464 | *MAST4* | A/G | A | 2.21 | (1.39, 3.52) | 1.04 | (0.61, 1.79) | 1.05 | (0.78, 1.42) | 0.50 | (0.16, 0.84) | 3.80E-03 | 7.35E-03 | rs17281154 | 1 |
| rs17245874 | 5 | 81527262 | *ATG10* | A/G | A | 1.76 | (1.2, 2.6) | 1.05 | (0.67, 1.63) | 1.01 | (0.72, 1.4) | 0.40 | (0.06, 0.75) | 2.21E-02 | 4.27E-02 | rs17245874 | 1 |
| rs3792794 | 5 | 150434722 | *TNIP1* | A/G | A | 2.22 | (1.39, 3.53) | 1.09 | (0.65, 1.85) | 1.06 | (0.79, 1.44) | 0.48 | (0.13, 0.83) | 7.11E-03 | 1.38E-02 | rs3805431 | 0.995 |
| rs3805431 | 5 | 150439539 | *TNIP1* | A/G | A | 2.23 | (1.4, 3.55) | 1.10 | (0.65, 1.86) | 1.06 | (0.79, 1.44) | 0.48 | (0.13, 0.83) | 6.93E-03 | 1.34E-02 | rs3805431 | 1 |
| rs4615314 | 5 | 168141366 | *SLIT3* | G/A | A | 1.85 | (1.24, 2.77) | 1.04 | (0.66, 1.62) | 1.02 | (0.74, 1.4) | 0.43 | (0.09, 0.77) | 1.22E-02 | 2.36E-02 | rs6890724 | 0.985 |
| rs6890724 | 5 | 168164077 | *SLIT3* | G/A | A | 1.90 | (1.27, 2.84) | 1.05 | (0.67, 1.65) | 1.02 | (0.74, 1.4) | 0.44 | (0.1, 0.77) | 1.03E-02 | 1.99E-02 | rs6890724 | 1 |
| rs17140294 | 6 | 5374744 | *FARS2* | G/A | G | 2.27 | (1.36, 3.8) | 1.00 | (0.53, 1.91) | 1.08 | (0.81, 1.45) | 0.52 | (0.15, 0.9) | 6.08E-03 | 1.18E-02 | rs17140294 | 1 |
| rs482393 | 6 | 7143604 | *RREB1* | G/A | G | 1.93 | (1.29, 2.89) | 1.11 | (0.69, 1.77) | 1.02 | (0.74, 1.41) | 0.41 | (0.06, 0.76) | 2.03E-02 | 3.92E-02 | rs482393 | 1 |
| rs34093877 | 6 | 106513510 | *21kb 5' of PRDM1* | G/A | G | 4.27 | (2.32, 7.89) | 1.70 | (0.8, 3.65) | 1.10 | (0.83, 1.47) | 0.58 | (0.19, 0.96) | 3.38E-03 | 6.54E-03 | rs34093877 | 1 |
| rs2898246 | 8 | 10656291 | *PINX1* | C/A | C | 2.35 | (1.31, 4.19) | 1.01 | (0.51, 1.99) | 1.11 | (0.83, 1.47) | 0.53 | (0.13, 0.92) | 9.27E-03 | 1.79E-02 | rs17152442 | 0.994 |
| rs73529608 | 8 | 10656486 | *PINX1* | T/A | T | 2.36 | (1.32, 4.21) | 1.01 | (0.51, 1.99) | 1.11 | (0.83, 1.48) | 0.53 | (0.13, 0.92) | 8.91E-03 | 1.72E-02 | rs17152442 | 0.994 |
| rs17152442 | 8 | 10657726 | *PINX1* | G/C | G | 2.35 | (1.31, 4.2) | 1.00 | (0.51, 1.98) | 1.10 | (0.83, 1.47) | 0.53 | (0.14, 0.92) | 8.16E-03 | 1.58E-02 | rs17152442 | 1 |
| rs17152632 | 8 | 10727072 | *27kb 3' of XKR6* | G/A | G | 2.43 | (1.34, 4.39) | 1.07 | (0.52, 2.21) | 1.11 | (0.84, 1.48) | 0.51 | (0.09, 0.94) | 1.77E-02 | 3.42E-02 | rs17152442 | 0.813 |
| rs1060242 | 8 | 67380528 | *ADHFE1* | G/A | A | 2.06 | (1.36, 3.11) | 1.03 | (0.64, 1.67) | 1.00 | (0.73, 1.37) | 0.50 | (0.18, 0.81) | 1.96E-03 | 3.78E-03 | rs1060242 | 1 |
| rs4742061 | 9 | 4978816 | *6.4kb 5' of JAK2* | G/A | G | 4.00 | (2.09, 7.66) | 1.76 | (0.89, 3.49) | 1.15 | (0.86, 1.53) | 0.52 | (0.11, 0.94) | 1.41E-02 | 2.73E-02 | rs4742061 | 1 |
| rs1072502 | 9 | 93914857 | *61kb 3' of AUH* | T/A | T | 1.98 | (1.27, 3.09) | 1.08 | (0.67, 1.76) | 1.07 | (0.78, 1.45) | 0.42 | (0.06, 0.78) | 2.37E-02 | 4.56E-02 | rs1072502 | 1 |
| rs74156431 | 10 | 61952950 | *ANK3* | T/A | T | 2.11 | (1.24, 3.59) | 1.04 | (0.59, 1.83) | 1.10 | (0.82, 1.48) | 0.46 | (0.06, 0.86) | 2.29E-02 | 4.41E-02 | rs74156431 | 1 |
| rs74156432 | 10 | 61953034 | *ANK3* | A/C | A | 2.11 | (1.24, 3.59) | 1.04 | (0.59, 1.83) | 1.11 | (0.82, 1.48) | 0.46 | (0.06, 0.86) | 2.31E-02 | 4.44E-02 | rs74156431 | 1 |
| rs7071579 | 10 | 81910278 | *4.6kb 3' of ANXA11* | G/A | A | 2.32 | (1.54, 3.49) | 1.26 | (0.81, 1.96) | 1.02 | (0.74, 1.41) | 0.45 | (0.13, 0.76) | 4.99E-03 | 9.66E-03 | rs7071579 | 1 |
| rs3759321 | 12 | 6062878 | *VWF* | A/G | G | 1.96 | (1.29, 2.96) | 1.01 | (0.63, 1.61) | 1.01 | (0.74, 1.39) | 0.48 | (0.16, 0.8) | 3.49E-03 | 6.75E-03 | rs3759321 | 1 |
| rs11057405 | 12 | 122781897 | *CLIP1* | A/G | A | 1.86 | (1.21, 2.85) | 1.03 | (0.63, 1.67) | 1.05 | (0.77, 1.43) | 0.42 | (0.05, 0.78) | 2.47E-02 | 4.75E-02 | rs11057405 | 1 |
| rs17612850 | 13 | 59591041 | *649kb 3' of DIAPH3* | A/G | A | 2.40 | (1.35, 4.28) | 1.11 | (0.6, 2.06) | 1.12 | (0.84, 1.49) | 0.49 | (0.08, 0.89) | 1.80E-02 | 3.47E-02 | rs17612850 | 1 |
| rs1570541 | 13 | 76074865 | *19kb 5' of TBC1D4* | G/A | G | 2.13 | (1.35, 3.36) | 1.08 | (0.62, 1.9) | 1.06 | (0.78, 1.43) | 0.46 | (0.09, 0.83) | 1.44E-02 | 2.78E-02 | rs6562898 | 1 |
| rs6562898 | 13 | 76076368 | *20kb 5' of TBC1D4* | A/G | A | 2.13 | (1.35, 3.36) | 1.09 | (0.62, 1.9) | 1.05 | (0.77, 1.41) | 0.47 | (0.1, 0.84) | 1.34E-02 | 2.58E-02 | rs6562898 | 1 |
| rs34700794 | 13 | 77663138 | *MYCBP2* | A/G | A | 2.57 | (1.51, 4.4) | 1.09 | (0.55, 2.17) | 1.10 | (0.82, 1.47) | 0.54 | (0.16, 0.92) | 5.48E-03 | 1.06E-02 | rs34700794 | 1 |
| rs72700301 | 14 | 98390882 | *1.1kb 3' of C14orf64* | A/G | A | 3.16 | (1.56, 6.42) | 1.20 | (0.56, 2.55) | 1.13 | (0.86, 1.5) | 0.58 | (0.17, 0.99) | 5.50E-03 | 1.06E-02 | rs72700301 | 1 |
| rs79128613 | 16 | 30788236 | *607bp 3' of RNF40* | G/A | G | 2.16 | (1.3, 3.59) | 1.09 | (0.63, 1.91) | 1.10 | (0.82, 1.48) | 0.45 | (0.05, 0.84) | 2.57E-02 | 4.95E-02 | rs78985803 | 0.972 |
| rs78985803 | 16 | 30812608 | *14kb 5' of ZNF629* | A/G | A | 2.18 | (1.31, 3.63) | 1.06 | (0.61, 1.85) | 1.06 | (0.79, 1.43) | 0.49 | (0.11, 0.86) | 1.07E-02 | 2.07E-02 | rs78985803 | 1 |
| rs11653290 | 17 | 6983529 | *CLEC10A* | C/A | C | 2.51 | (1.61, 3.9) | 1.37 | (0.81, 2.34) | 1.05 | (0.77, 1.42) | 0.43 | (0.07, 0.8) | 2.03E-02 | 3.90E-02 | rs11653290 | 1 |
| rs2290065 | 17 | 38716360 | *CCR7* | A/G | A | 2.37 | (1.41, 3.97) | 1.19 | (0.66, 2.14) | 1.10 | (0.82, 1.48) | 0.46 | (0.06, 0.85) | 2.49E-02 | 4.79E-02 | rs2290065 | 1 |
| rs17072300 | 18 | 61631433 | *3.8kb 3' of HMSD* | A/G | A | 2.51 | (1.43, 4.41) | 1.16 | (0.59, 2.26) | 1.10 | (0.83, 1.47) | 0.50 | (0.09, 0.91) | 1.75E-02 | 3.38E-02 | rs17072300 | 1 |
| rs73055288 | 19 | 55450462 | *NLRP7* | A/C | A | 2.35 | (1.42, 3.9) | 1.05 | (0.56, 1.96) | 1.08 | (0.8, 1.44) | 0.52 | (0.15, 0.89) | 5.33E-03 | 1.03E-02 | rs73055288 | 1 |
| rs34400411 | 21 | 45596710 | *31kb 3' of C21orf33* | C/A | C | 2.12 | (1.32, 3.39) | 1.06 | (0.64, 1.77) | 1.07 | (0.79, 1.45) | 0.46 | (0.11, 0.82) | 1.04E-02 | 2.01E-02 | rs34400411 | 1 |

**TABLE S2.** Summary of findings from gene-environment interaction between cigarette smoking in non-LS at FDR < 0.05

| Marker name | CHR | sition (hg19) | Marker location w RefSeq Gene | Minor/Major alleles | coded allele | $OR_{SNP-SMOK}$ | 95%CI | $OR_{SNP}$ | 95%CI | $OR_{SMOK}$ | 95%CI | AP | 95%CI | AP p-val | FDR | Best tag SNP | LD $r^2$ |
|---|---|---|---|---|---|---|---|---|---|---|---|---|---|---|---|---|---|
| rs12565567 | 1 | 67651412 | *IL23R* | A/G | A | 3.05 | (1.69, 5.5) | 1.20 | (0.63, 2.28) | 1.05 | (0.84, 1.31) | 0.59 | (0.24, 0.94) | 8.24E-04 | 1.88E-03 | rs12565567 | 1 |
| rs17129698 | 1 | 67654072 | *IL23R* | A/G | A | 3.05 | (1.69, 5.5) | 1.22 | (0.64, 2.32) | 1.05 | (0.84, 1.31) | 0.58 | (0.23, 0.94) | 1.13E-03 | 2.57E-03 | rs12565567 | 0.995 |
| rs61780310 | 1 | 67659353 | *IL23R* | G/A | G | 3.03 | (1.68, 5.45) | 1.19 | (0.63, 2.27) | 1.05 | (0.84, 1.31) | 0.59 | (0.24, 0.94) | 8.56E-04 | 1.95E-03 | rs12565567 | 0.985 |
| rs61780311 | 1 | 67661307 | *IL23R* | G/C | G | 3.24 | (1.75, 5.97) | 1.21 | (0.62, 2.37) | 1.05 | (0.84, 1.31) | 0.61 | (0.27, 0.95) | 4.94E-04 | 1.13E-03 | rs12565567 | 0.839 |
| rs61780312 | 1 | 67661648 | *IL23R* | C/A | C | 3.23 | (1.75, 5.97) | 1.18 | (0.6, 2.31) | 1.05 | (0.84, 1.31) | 0.62 | (0.28, 0.95) | 3.00E-04 | 6.85E-04 | rs12565567 | 0.840 |
| rs28464018 | 1 | 67662430 | *IL23R* | G/A | G | 3.23 | (1.75, 5.97) | 1.19 | (0.61, 2.32) | 1.05 | (0.84, 1.31) | 0.62 | (0.28, 0.95) | 3.31E-04 | 7.54E-04 | rs12565567 | 0.845 |
| rs116482055 | 1 | 101263357 | *59kb 3' of VCAM1* | A/G | A | 2.21 | (1.33, 3.68) | 1.12 | (0.64, 1.97) | 1.05 | (0.84, 1.31) | 0.47 | (0.08, 0.86) | 1.87E-02 | 4.23E-02 | rs116482055 | 1 |
| rs7554873 | 1 | 161612233 | *11kb 5' of FCGR3B* | G/A | G | 2.00 | (1.29, 3.1) | 1.09 | (0.66, 1.79) | 1.03 | (0.82, 1.3) | 0.44 | (0.08, 0.8) | 1.72E-02 | 3.89E-02 | rs7554873 | 1 |
| rs114384494 | 1 | 161653554 | *RPL31P11* | A/G | A | 2.16 | (1.35, 3.44) | 1.09 | (0.63, 1.89) | 1.03 | (0.82, 1.3) | 0.48 | (0.11, 0.85) | 1.04E-02 | 2.36E-02 | rs114384494 | 1 |
| rs12760697 | 1 | 192416200 | *80kb 3' of RGS21* | A/G | A | 2.84 | (1.5, 5.35) | 1.29 | (0.67, 2.49) | 1.07 | (0.85, 1.33) | 0.52 | (0.1, 0.94) | 1.49E-02 | 3.39E-02 | rs12760697 | 1 |
| rs410607 | 2 | 185445880 | *17kb 5' of ZNF804A* | A/G | A | 2.42 | (1.36, 4.32) | 1.11 | (0.55, 2.22) | 1.05 | (0.84, 1.32) | 0.52 | (0.1, 0.94) | 1.49E-02 | 3.39E-02 | rs410607 | 1 |
| rs359900 | 2 | 185451906 | *11kb 5' of ZNF804A* | A/G | A | 2.42 | (1.36, 4.32) | 1.11 | (0.55, 2.22) | 1.05 | (0.84, 1.32) | 0.52 | (0.1, 0.94) | 1.49E-02 | 3.39E-02 | rs410607 | 1 |
| rs359901 | 2 | 185452728 | *10kb 5' of ZNF804A* | A/G | A | 2.42 | (1.36, 4.32) | 1.11 | (0.55, 2.22) | 1.05 | (0.84, 1.32) | 0.52 | (0.1, 0.94) | 1.49E-02 | 3.39E-02 | rs410607 | 1 |
| rs35272229 | 2 | 197946392 | *ANKRD44* | A/G | A | 1.82 | (1.17, 2.84) | 1.01 | (0.65, 1.56) | 1.04 | (0.82, 1.31) | 0.43 | (0.07, 0.78) | 1.77E-02 | 4.01E-02 | rs35272229 | 1 |
| rs13168857 | 5 | 10676692 | *2.6kb 3' of DAP* | C/A | C | 2.24 | (1.22, 4.11) | 1.01 | (0.52, 1.95) | 1.07 | (0.86, 1.34) | 0.52 | (0.1, 0.93) | 1.51E-02 | 3.42E-02 | rs13168857 | 1 |
| rs17245714 | 5 | 34998894 | *AGXT2* | C/G | C | 1.95 | (1.29, 2.94) | 1.07 | (0.68, 1.66) | 1.02 | (0.81, 1.29) | 0.44 | (0.11, 0.78) | 8.93E-03 | 2.03E-02 | rs17245714 | 1 |
| rs2106491 | 5 | 135348376 | *16kb 5' of TGFBI* | A/G | G | 2.21 | (1.27, 3.83) | 1.03 | (0.55, 1.92) | 1.05 | (0.84, 1.31) | 0.51 | (0.12, 0.91) | 1.12E-02 | 2.55E-02 | rs2106491 | 1 |
| rs17658318 | 5 | 150322270 | *ZNF300P1* | A/G | A | 1.99 | (1.32, 2.99) | 1.05 | (0.69, 1.61) | 1.01 | (0.8, 1.28) | 0.47 | (0.15, 0.78) | 3.47E-03 | 7.92E-03 | rs17658318 | 1 |
| rs10485038 | 6 | 90814274 | *BACH2* | G/C | G | 3.24 | (1.87, 5.61) | 1.50 | (0.87, 2.59) | 1.05 | (0.84, 1.32) | 0.52 | (0.16, 0.88) | 4.13E-03 | 9.42E-03 | rs57858238 | 0.995 |
| rs207258 | 6 | 90814496 | *BACH2* | C/G | C | 2.29 | (1.55, 3.36) | 1.33 | (0.88, 1.99) | 1.00 | (0.79, 1.27) | 0.42 | (0.1, 0.73) | 9.01E-03 | 2.05E-02 | rs207258 | 1 |
| rs57858238 | 6 | 90820248 | *BACH2* | A/G | A | 3.32 | (1.91, 5.76) | 1.50 | (0.87, 2.59) | 1.05 | (0.84, 1.32) | 0.53 | (0.18, 0.88) | 2.79E-03 | 6.37E-03 | rs57858238 | 1 |
| rs10498966 | 6 | 91032094 | *9.5kb 3' of MIR4464* | G/A | G | 2.36 | (1.39, 4.01) | 1.02 | (0.58, 1.81) | 1.04 | (0.83, 1.3) | 0.55 | (0.21, 0.89) | 1.53E-03 | 3.49E-03 | rs10498966 | 1 |
| rs62427027 | 6 | 126491161 | *131kb 3' of TRMT11* | A/G | A | 1.82 | (1.2, 2.75) | 1.04 | (0.68, 1.6) | 1.03 | (0.81, 1.3) | 0.41 | (0.07, 0.76) | 1.81E-02 | 4.10E-02 | rs62427027 | 1 |
| rs2724099 | 7 | 93435670 | *80kb 3' of TFPI2* | G/A | G | 1.72 | (1.18, 2.5) | 1.03 | (0.67, 1.58) | 1.01 | (0.8, 1.28) | 0.40 | (0.06, 0.74) | 2.21E-02 | 4.99E-02 | rs2724099 | 1 |
| rs74152075 | 10 | 101329082 | *33kb 3' of NKX2-3* | A/G | A | 2.02 | (1.2, 3.42) | 1.00 | (0.57, 1.75) | 1.05 | (0.84, 1.32) | 0.48 | (0.09, 0.87) | 1.53E-02 | 3.47E-02 | rs74152075 | 1 |
| rs118042921 | 11 | 579736 | *PHRF1* | G/A | G | 2.36 | (1.3, 4.3) | 1.01 | (0.5, 2.04) | 1.06 | (0.85, 1.32) | 0.55 | (0.15, 0.95) | 7.76E-03 | 1.77E-02 | rs118042921 | 1 |
| rs61729556 | 11 | 7694002 | *CYB5R2* | A/G | A | 2.29 | (1.44, 3.64) | 1.10 | (0.61, 1.96) | 1.03 | (0.82, 1.29) | 0.51 | (0.15, 0.87) | 5.31E-03 | 1.21E-02 | rs61729556 | 1 |
| rs79460410 | 11 | 60777914 | *CD6* | A/G | A | 2.81 | (1.47, 5.37) | 1.01 | (0.51, 2.01) | 1.06 | (0.85, 1.32) | 0.62 | (0.27, 0.97) | 5.19E-04 | 1.19E-03 | rs79460410 | 1 |
| rs177372 | 14 | 73757745 | *NUMB* | A/G | A | 2.54 | (1.31, 4.94) | 1.05 | (0.52, 2.1) | 1.07 | (0.85, 1.33) | 0.56 | (0.15, 0.97) | 6.84E-03 | 1.56E-02 | rs177372 | 1 |
| rs177369 | 14 | 73760442 | *NUMB* | G/A | G | 2.51 | (1.29, 4.86) | 1.04 | (0.52, 2.07) | 1.07 | (0.85, 1.33) | 0.56 | (0.15, 0.97) | 7.08E-03 | 1.61E-02 | rs177372 | 0.988 |
| rs5479 | 16 | 67469733 | *HSD11B2* | A/C | A | 2.35 | (1.4, 3.93) | 1.09 | (0.6, 1.95) | 1.04 | (0.83, 1.31) | 0.52 | (0.15, 0.88) | 5.32E-03 | 1.21E-02 | rs75204333 | 0.917 |
| rs75204333 | 16 | 67574408 | *FAM65A* | G/A | G | 2.30 | (1.39, 3.8) | 1.02 | (0.57, 1.82) | 1.03 | (0.83, 1.3) | 0.54 | (0.2, 0.89) | 2.09E-03 | 4.78E-03 | rs75204333 | 1 |
| rs4987801 | 18 | 60845099 | *BCL2* | G/A | G | 2.04 | (1.32, 3.16) | 1.13 | (0.71, 1.79) | 1.03 | (0.82, 1.3) | 0.43 | (0.08, 0.78) | 1.50E-02 | 3.40E-02 | rs4987801 | 1 |
| rs229495 | 22 | 37563388 | *13kb 3' of C1QTNF6* | A/G | A | 2.16 | (1.27, 3.68) | 1.01 | (0.52, 1.93) | 1.05 | (0.84, 1.31) | 0.51 | (0.11, 0.91) | 1.18E-02 | 2.69E-02 | rs229495 | 1 |

**TABLE S3.** Summary of findings from gene-environment interaction between cigarette smoking in LS after adding adjustment for HLA-DRB1*03 at FDR < 0.05

| Marker name | CHR | Position | Marker location w RefSeq Gene | Minor/Major alleles | coded allele | $OR_{SNP-SMOK}$ | 95%CI | $OR_{SNP}$ | 95%CI | $OR_{SMOK}$ | 95%CI | AP | 95%CI | AP p-val | FDR | Best tag SNP | LD $r^2$ |
|---|---|---|---|---|---|---|---|---|---|---|---|---|---|---|---|---|---|
| rs6681938 | 1 | 1781220 | *GNB1* | G/A | G | 1.77 | (1.17, 2.66) | 1.08 | (0.71, 1.64) | 1.00 | (0.66, 1.52) | 0.39 | (0.05, 0.73) | 2.52E-02 | 4.86E-02 | rs6681938 | 1.000 |
| rs2227313 | 1 | 2487766 | *LOC100133445* | G/A | A | 2.13 | (1.37, 3.33) | 1.03 | (0.63, 1.71) | 1.07 | (0.77, 1.5) | 0.48 | (0.15, 0.81) | 4.36E-03 | 8.43E-03 | rs2227313 | 1.000 |
| rs781081904 | 1 | 19331408 | *49kb 5' of IFFO2* | G/A | G | 2.04 | (1.3, 3.19) | 1.07 | (0.63, 1.81) | 1.10 | (0.79, 1.53) | 0.43 | (0.05, 0.8) | 2.47E-02 | 4.75E-02 | rs1078895 | 1.000 |
| **rs12117581** | 1 | 25272809 | *RUNX3* | A/G | A | 2.29 | (1.34, 3.93) | 1.02 | (0.56, 1.86) | 1.14 | (0.84, 1.57) | 0.49 | (0.11, 0.87) | 1.10E-02 | 2.12E-02 | rs12117581 | 1.000 |
| rs11206127 | 1 | 53713549 | *LRP8* | A/G | G | 2.32 | (1.48, 3.62) | 1.05 | (0.64, 1.73) | 1.05 | (0.75, 1.47) | 0.52 | (0.22, 0.83) | 8.00E-04 | 1.55E-03 | rs3737983 | 0.997 |
| rs3737983 | 1 | 53716416 | *LRP8* | A/G | G | 2.31 | (1.48, 3.62) | 1.03 | (0.62, 1.7) | 1.05 | (0.75, 1.47) | 0.53 | (0.23, 0.84) | 5.59E-04 | 1.08E-03 | rs3737983 | 1.000 |
| rs869987 | 1 | 53723190 | *LRP8* | A/C | C | 2.24 | (1.43, 3.51) | 1.03 | (0.62, 1.69) | 1.05 | (0.75, 1.47) | 0.52 | (0.2, 0.83) | 1.30E-03 | 2.51E-03 | rs3737983 | 0.992 |
| rs869988 | 1 | 53723349 | *LRP8* | G/A | A | 2.32 | (1.48, 3.63) | 1.05 | (0.64, 1.73) | 1.05 | (0.75, 1.47) | 0.52 | (0.22, 0.83) | 7.99E-04 | 1.55E-03 | rs3737983 | 0.994 |
| rs6668172 | 1 | 53729210 | *LRP8* | A/G | G | 2.32 | (1.49, 3.63) | 1.05 | (0.64, 1.73) | 1.05 | (0.75, 1.47) | 0.53 | (0.22, 0.83) | 7.52E-04 | 1.45E-03 | rs3737983 | 0.995 |
| rs7526226 | 1 | 53731019 | *LRP8* | A/T | T | 2.31 | (1.48, 3.62) | 1.09 | (0.66, 1.79) | 1.05 | (0.75, 1.47) | 0.51 | (0.19, 0.82) | 1.60E-03 | 3.10E-03 | rs3737983 | 0.995 |
| rs7528745 | 1 | 53731265 | *LRP8* | A/T | T | 2.32 | (1.49, 3.64) | 1.05 | (0.64, 1.73) | 1.05 | (0.75, 1.47) | 0.53 | (0.22, 0.83) | 7.53E-04 | 1.46E-03 | rs3737983 | 0.995 |
| rs10888778 | 1 | 53735446 | *LRP8* | A/C | C | 2.33 | (1.49, 3.65) | 1.06 | (0.64, 1.74) | 1.05 | (0.75, 1.47) | 0.52 | (0.22, 0.83) | 8.33E-04 | 1.61E-03 | rs3737983 | 0.994 |
| rs11206132 | 1 | 53739185 | *LRP8* | A/G | G | 2.33 | (1.49, 3.64) | 1.05 | (0.64, 1.73) | 1.05 | (0.75, 1.47) | 0.52 | (0.22, 0.83) | 7.74E-04 | 1.50E-03 | rs3737983 | 0.994 |
| **rs12132140** | 1 | 157767362 | *FCRL1* | A/G | A | 3.36 | (1.87, 6.04) | 1.08 | (0.56, 2.12) | 1.13 | (0.83, 1.54) | 0.64 | (0.34, 0.94) | 2.86E-05 | 5.53E-05 | rs12132140 | 1.000 |
| rs72739794 | 1 | 198912668 | *6.1kb 5' of LOC100131234* | G/A | G | 3.10 | (1.52, 6.33) | 1.22 | (0.56, 2.66) | 1.20 | (0.89, 1.62) | 0.54 | (0.1, 0.98) | 1.61E-02 | 3.11E-02 | rs72739794 | 1.000 |
| **rs115265657** | 1 | 200549451 | *KIF14* | G/A | G | 3.62 | (1.73, 7.58) | 1.27 | (0.56, 2.86) | 1.19 | (0.88, 1.61) | 0.60 | (0.19, 1) | 3.83E-03 | 7.41E-03 | rs115265657 | 1.000 |
| rs1795033 | 1 | 214293994 | *84kb 3' of PROX1* | C/A | C | 2.78 | (1.64, 4.72) | 1.38 | (0.77, 2.48) | 1.16 | (0.84, 1.59) | 0.45 | (0.05, 0.84) | 2.59E-02 | 4.99E-02 | rs1795033 | 1.000 |
| **rs12083512** | 1 | 215057759 | *121kb 5' of KCNK2* | G/A | G | 2.91 | (1.52, 5.54) | 1.14 | (0.56, 2.3) | 1.18 | (0.87, 1.6) | 0.55 | (0.15, 0.94) | 6.80E-03 | 1.32E-02 | rs12083512 | 1.000 |
| rs74374206 | 2 | 100815926 | *57kb 5' of AFF3* | G/A | G | 2.08 | (1.26, 3.43) | 1.01 | (0.57, 1.81) | 1.13 | (0.82, 1.56) | 0.45 | (0.05, 0.84) | 2.59E-02 | 4.98E-02 | rs114351924 | 1.000 |
| rs114351924 | 2 | 100818235 | *59kb 5' of AFF3* | A/G | A | 2.08 | (1.26, 3.43) | 1.01 | (0.57, 1.81) | 1.13 | (0.82, 1.56) | 0.45 | (0.05, 0.84) | 2.58E-02 | 4.97E-02 | rs114351924 | 1.000 |
| rs6725016 | 2 | 106124178 | *69kb 5' of FHL2* | C/A | C | 1.80 | (1.21, 2.68) | 1.01 | (0.65, 1.58) | 1.02 | (0.71, 1.46) | 0.43 | (0.09, 0.77) | 1.27E-02 | 2.45E-02 | rs6725016 | 1.000 |
| rs7558231 | 2 | 133591129 | *NCKAP5* | A/G | A | 2.65 | (1.54, 4.54) | 1.18 | (0.61, 2.27) | 1.11 | (0.81, 1.52) | 0.51 | (0.13, 0.9) | 8.91E-03 | 1.72E-02 | rs7558231 | 1.000 |
| rs201908473 | 2 | 136863543 | *8.4kb 3' of CXCR4* | A/C | A | 1.96 | (1.29, 2.99) | 1.13 | (0.72, 1.75) | 1.06 | (0.74, 1.53) | 0.39 | (0.05, 0.74) | 2.45E-02 | 4.72E-02 | rs201908473 | 1.000 |
| rs1869325 | 2 | 138084574 | *THSD7B* | G/C | G | 2.34 | (1.31, 4.19) | 1.05 | (0.58, 1.91) | 1.17 | (0.86, 1.6) | 0.48 | (0.08, 0.88) | 1.93E-02 | 3.72E-02 | rs1869325 | 1.000 |
| **rs62198467** | 2 | 185804581 | *366bp 3' of ZNF804A* | G/A | G | 2.82 | (1.71, 4.66) | 1.11 | (0.65, 1.9) | 1.09 | (0.79, 1.5) | 0.57 | (0.28, 0.87) | 1.35E-04 | 2.61E-04 | rs62198467 | 1.000 |
| rs17720776 | 2 | 194214645 | *573kb 3' of PCGEM1* | G/A | G | 2.40 | (1.46, 3.96) | 1.15 | (0.61, 2.16) | 1.13 | (0.82, 1.54) | 0.47 | (0.08, 0.87) | 1.92E-02 | 3.71E-02 | rs17720776 | 1.000 |
| rs1367311 | 3 | 156418756 | *TIPARP* | A/G | A | 1.98 | (1.3, 3) | 1.02 | (0.64, 1.64) | 1.04 | (0.73, 1.47) | 0.46 | (0.13, 0.79) | 5.98E-03 | 1.16E-02 | rs1367311 | 1.000 |
| rs7657055 | 4 | 6311478 | *6.5kb 3' of WFS1* | G/C | G | 2.60 | (1.35, 5.03) | 1.11 | (0.55, 2.23) | 1.19 | (0.88, 1.62) | 0.50 | (0.06, 0.94) | 2.52E-02 | 4.85E-02 | rs7657055 | 1.000 |
| rs17001561 | 4 | 77096118 | *SCARB2* | A/G | A | 2.18 | (1.4, 3.39) | 1.04 | (0.64, 1.69) | 1.06 | (0.75, 1.49) | 0.50 | (0.18, 0.82) | 2.08E-03 | 4.02E-03 | rs17001561 | 1.000 |
| rs72655564 | 4 | 77169876 | *3kb 5' of FAM47E* | C/A | C | 2.32 | (1.38, 3.88) | 1.07 | (0.61, 1.89) | 1.15 | (0.84, 1.58) | 0.47 | (0.1, 0.85) | 1.29E-02 | 2.49E-02 | rs72655564 | 1.000 |
| rs1399405 | 4 | 108486838 | *48kb 3' of PAPSS1* | C/G | G | 2.16 | (1.41, 3.33) | 1.11 | (0.68, 1.82) | 1.06 | (0.75, 1.49) | 0.46 | (0.12, 0.79) | 7.49E-03 | 1.45E-02 | rs2726686 | 0.975 |
| rs2672477 | 4 | 108498781 | *36kb 3' of PAPSS1* | G/A | A | 2.14 | (1.39, 3.28) | 1.09 | (0.67, 1.78) | 1.06 | (0.75, 1.48) | 0.46 | (0.13, 0.8) | 6.23E-03 | 1.20E-02 | rs2726686 | 0.999 |
| rs2726686 | 4 | 108499056 | *36kb 3' of PAPSS1* | G/A | A | 2.14 | (1.39, 3.28) | 1.09 | (0.67, 1.78) | 1.06 | (0.75, 1.48) | 0.46 | (0.13, 0.8) | 6.22E-03 | 1.20E-02 | rs2726686 | 1.000 |
| **rs7665274** | 4 | 108690915 | *49kb 5' of PAPSS1* | G/A | G | 1.90 | (1.24, 2.92) | 1.03 | (0.64, 1.66) | 1.08 | (0.76, 1.52) | 0.42 | (0.06, 0.77) | 2.14E-02 | 4.12E-02 | rs7665274 | 1.000 |
| rs62321881 | 4 | 122932994 | *60kb 5' of TRPC3* | G/A | G | 2.56 | (1.44, 4.55) | 1.09 | (0.57, 2.07) | 1.16 | (0.85, 1.58) | 0.51 | (0.12, 0.9) | 9.85E-03 | 1.90E-02 | rs62321881 | 1.000 |
| rs1472328 | 4 | 176644821 | *GPM6A* | G/A | G | 2.19 | (1.39, 3.44) | 1.03 | (0.62, 1.7) | 1.07 | (0.77, 1.5) | 0.50 | (0.17, 0.82) | 2.68E-03 | 5.18E-03 | rs1472328 | 1.000 |
| rs6881582 | 5 | 14089644 | *54kb 5' of TRIO* | A/G | A | 1.77 | (1.18, 2.66) | 1.02 | (0.67, 1.56) | 1.01 | (0.69, 1.48) | 0.42 | (0.08, 0.75) | 1.43E-02 | 2.76E-02 | rs6881582 | 1.000 |
| rs11948370 | 5 | 30726054 | *468kb 5' of CDH6* | G/A | G | 2.19 | (1.33, 3.62) | 1.00 | (0.55, 1.83) | 1.12 | (0.82, 1.54) | 0.49 | (0.11, 0.86) | 1.12E-02 | 2.16E-02 | rs11948370 | 1.000 |
| rs12655342 | 5 | 96229542 | *ERAP2* | C/A | C | 1.98 | (1.29, 3.06) | 1.06 | (0.67, 1.68) | 1.08 | (0.77, 1.53) | 0.42 | (0.08, 0.77) | 1.54E-02 | 2.97E-02 | rs12655342 | 1.000 |
| **rs3792794** | 5 | 150434722 | *TNIP1* | A/G | A | 2.29 | (1.4, 3.76) | 1.06 | (0.61, 1.87) | 1.12 | (0.81, 1.54) | 0.48 | (0.12, 0.84) | 8.73E-03 | 1.69E-02 | rs3805431 | 0.995 |
| **rs3805431** | 5 | 150439539 | *TNIP1* | A/G | A | 2.30 | (1.4, 3.77) | 1.07 | (0.61, 1.87) | 1.12 | (0.81, 1.54) | 0.48 | (0.12, 0.84) | 8.57E-03 | 1.66E-02 | rs3805431 | 1.000 |
| **rs4615314** | 5 | 168141366 | *SLIT3* | G/A | A | 2.24 | (1.45, 3.47) | 1.17 | (0.73, 1.89) | 1.08 | (0.77, 1.52) | 0.44 | (0.1, 0.78) | 1.02E-02 | 1.97E-02 | rs6890724 | 0.985 |
| **rs6890724** | 5 | 168164077 | *SLIT3* | G/A | A | 2.30 | (1.48, 3.57) | 1.18 | (0.73, 1.91) | 1.08 | (0.77, 1.51) | 0.45 | (0.12, 0.78) | 7.04E-03 | 1.36E-02 | rs6890724 | 1.000 |
| rs6889721 | 5 | 171513431 | *STK10* | G/C | G | 2.07 | (1.35, 3.17) | 1.06 | (0.66, 1.68) | 1.04 | (0.74, 1.48) | 0.47 | (0.15, 0.79) | 4.42E-03 | 8.56E-03 | rs6889721 | 1.000 |
| rs4959782 | 6 | 3252135 | *7kb 5' of PSMG4* | C/A | C | 2.25 | (1.48, 3.41) | 1.12 | (0.71, 1.75) | 1.01 | (0.71, 1.44) | 0.50 | (0.2, 0.79) | 9.81E-04 | 1.90E-03 | rs4959782 | 1.000 |
| rs11242833 | 6 | 3255885 | *3.3kb 5' of PSMG4* | C/G | C | 2.14 | (1.39, 3.29) | 1.16 | (0.74, 1.82) | 1.08 | (0.76, 1.53) | 0.42 | (0.09, 0.75) | 1.30E-02 | 2.50E-02 | rs4959782 | 0.944 |
| rs4445096 | 6 | 3256895 | *2.3kb 5' of PSMG4* | A/G | A | 2.14 | (1.39, 3.29) | 1.16 | (0.74, 1.82) | 1.08 | (0.76, 1.53) | 0.42 | (0.09, 0.75) | 1.29E-02 | 2.49E-02 | rs4959782 | 0.943 |
| rs4959787 | 6 | 3264502 | *PSMG4* | G/C | G | 2.01 | (1.32, 3.07) | 1.12 | (0.71, 1.75) | 1.07 | (0.75, 1.53) | 0.41 | (0.07, 0.75) | 1.83E-02 | 3.54E-02 | rs4959782 | 0.864 |
| **rs482393** | 6 | 7143604 | *RREB1* | G/A | G | 2.18 | (1.42, 3.35) | 1.18 | (0.72, 1.94) | 1.08 | (0.77, 1.51) | 0.42 | (0.07, 0.77) | 1.83E-02 | 3.54E-02 | rs482393 | 1.000 |
| rs9296399 | 6 | 42778467 | *10kb 5' of KIAA0240* | G/A | G | 2.72 | (1.4, 5.28) | 1.11 | (0.54, 2.3) | 1.17 | (0.86, 1.58) | 0.53 | (0.1, 0.96) | 1.46E-02 | 2.82E-02 | rs9296399 | 1.000 |
| rs78756932 | 6 | 106606408 | *26kb 3' of ATG5* | A/C | A | 3.51 | (1.72, 7.2) | 1.17 | (0.55, 2.5) | 1.19 | (0.88, 1.61) | 0.61 | (0.24, 0.98) | 1.14E-03 | 2.21E-03 | rs78756932 | 1.000 |
| rs6557142 | 6 | 151784049 | *C6orf211* | A/C | A | 2.43 | (1.37, 4.3) | 1.03 | (0.51, 2.05) | 1.16 | (0.85, 1.58) | 0.51 | (0.11, 0.92) | 1.27E-02 | 2.46E-02 | rs17081270 | 1.000 |
| rs17081270 | 6 | 151785551 | *C6orf211* | G/A | G | 2.57 | (1.44, 4.55) | 1.05 | (0.52, 2.11) | 1.14 | (0.83, 1.55) | 0.54 | (0.15, 0.92) | 6.72E-03 | 1.30E-02 | rs17081270 | 1.000 |

**TABLE S3.** Summary of findings from gene-environment interaction between cigarette smoking in LS after adding adjustment for HLA-DRB1*03 at FDR < 0.05

| | | | | | | | | | | | | | | | | | |
|---|---|---|---|---|---|---|---|---|---|---|---|---|---|---|---|---|---|
| **rs2898246** | 8 | 10656291 | *PINX1* | C/A | C | 2.75 | (1.48, 5.1) | 1.06 | (0.51, 2.2) | 1.16 | (0.86, 1.58) | 0.55 | (0.17, 0.94) | 4.93E-03 | 9.54E-03 | rs73529608 | 0.994 |
| **rs73529608** | 8 | 10656486 | *PINX1* | T/A | T | 2.75 | (1.48, 5.11) | 1.06 | (0.51, 2.2) | 1.17 | (0.86, 1.58) | 0.56 | (0.17, 0.94) | 4.81E-03 | 9.30E-03 | rs73529608 | 0.994 |
| **rs17152442** | 8 | 10657726 | *PINX1* | G/C | G | 2.73 | (1.47, 5.08) | 1.01 | (0.48, 2.1) | 1.16 | (0.85, 1.57) | 0.57 | (0.2, 0.95) | 2.52E-03 | 4.87E-03 | rs17152442 | 1.000 |
| **rs17152632** | 8 | 10727072 | *27kb 3' of XKR6* | G/A | G | 2.87 | (1.53, 5.37) | 1.20 | (0.55, 2.61) | 1.18 | (0.87, 1.6) | 0.52 | (0.09, 0.95) | 1.90E-02 | 3.66E-02 | rs17152442 | 0.813 |
| rs75536426 | 8 | 10863221 | *XKR6* | C/G | C | 3.20 | (1.69, 6.04) | 1.28 | (0.66, 2.47) | 1.20 | (0.88, 1.63) | 0.54 | (0.16, 0.92) | 5.30E-03 | 1.02E-02 | rs75536426 | 1.000 |
| rs4840550 | 8 | 11029029 | *XKR6* | A/C | C | 2.38 | (1.34, 4.2) | 1.22 | (0.67, 2.21) | 1.10 | (0.78, 1.54) | 0.44 | (0.09, 0.8) | 1.32E-02 | 2.54E-02 | rs4840550 | 1.000 |
| rs10100187 | 8 | 11257495 | *C8orf12* | A/G | G | 2.88 | (1.58, 5.24) | 1.34 | (0.74, 2.44) | 1.16 | (0.84, 1.59) | 0.48 | (0.11, 0.85) | 1.13E-02 | 2.18E-02 | rs10100187 | 1.000 |
| rs2467519 | 8 | 11399011 | *BLK* | A/G | G | 2.10 | (1.2, 3.68) | 1.11 | (0.62, 2) | 1.10 | (0.79, 1.54) | 0.42 | (0.07, 0.78) | 1.98E-02 | 3.82E-02 | rs2467519 | 1.000 |
| rs11783137 | 8 | 11448738 | *9.9kb 3' of NCRNA00208* | A/C | A | 2.57 | (1.47, 4.49) | 1.21 | (0.66, 2.23) | 1.16 | (0.85, 1.58) | 0.47 | (0.07, 0.86) | 2.05E-02 | 3.95E-02 | rs11783137 | 1.000 |
| rs2013211 | 8 | 22433508 | *499bp 3' of SORBS3* | A/G | G | 2.26 | (1.32, 3.88) | 1.01 | (0.57, 1.8) | 1.15 | (0.84, 1.57) | 0.49 | (0.11, 0.86) | 1.09E-02 | 2.10E-02 | rs2013211 | 1.000 |
| **rs1060242** | 8 | 67380528 | *ADHFE1* | G/A | A | 2.11 | (1.36, 3.29) | 1.08 | (0.64, 1.79) | 1.08 | (0.78, 1.51) | 0.45 | (0.1, 0.8) | 1.15E-02 | 2.21E-02 | rs1060242 | 1.000 |
| rs7832689 | 8 | 71971471 | *138kb 3' of EYA1* | A/C | A | 1.92 | (1.24, 2.97) | 1.02 | (0.63, 1.65) | 1.09 | (0.77, 1.53) | 0.42 | (0.07, 0.78) | 2.02E-02 | 3.89E-02 | rs7832689 | 1.000 |
| **rs4742061** | 9 | 4978816 | *6.4kb 5' of JAK2* | G/A | G | 4.04 | (1.98, 8.23) | 1.50 | (0.72, 3.12) | 1.21 | (0.89, 1.64) | 0.58 | (0.18, 0.97) | 4.26E-03 | 8.24E-03 | rs4742061 | 1.000 |
| rs7030315 | 9 | 5020529 | *JAK2* | A/G | A | 2.55 | (1.27, 5.11) | 1.05 | (0.52, 2.09) | 1.20 | (0.88, 1.62) | 0.51 | (0.07, 0.95) | 2.23E-02 | 4.30E-02 | rs7030315 | 1.000 |
| **rs74156431** | 10 | 61952950 | *ANK3* | T/A | T | 2.41 | (1.38, 4.21) | 1.03 | (0.56, 1.89) | 1.15 | (0.84, 1.57) | 0.51 | (0.14, 0.88) | 7.26E-03 | 1.41E-02 | rs74156431 | 1.000 |
| **rs74156432** | 10 | 61953034 | *ANK3* | A/C | A | 2.41 | (1.38, 4.21) | 1.03 | (0.56, 1.89) | 1.15 | (0.84, 1.57) | 0.51 | (0.14, 0.88) | 7.31E-03 | 1.41E-02 | rs74156431 | 1.000 |
| **rs7071579** | 10 | 81910278 | *4.6kb 3' of ANXA11* | G/A | A | 2.60 | (1.68, 4.01) | 1.27 | (0.79, 2.03) | 1.05 | (0.74, 1.48) | 0.49 | (0.19, 0.79) | 1.36E-03 | 2.63E-03 | rs7071579 | 1.000 |
| rs1332102 | 10 | 101324436 | *28kb 3' of NKX2-3* | T/A | A | 2.59 | (1.38, 4.88) | 1.03 | (0.49, 2.16) | 1.17 | (0.87, 1.59) | 0.54 | (0.12, 0.95) | 1.09E-02 | 2.11E-02 | rs1332102 | 1.000 |
| rs11200423 | 10 | 123891773 | *TACC2* | C/G | C | 2.63 | (1.33, 5.2) | 1.04 | (0.46, 2.35) | 1.19 | (0.88, 1.61) | 0.53 | (0.08, 0.98) | 2.01E-02 | 3.87E-02 | rs11200423 | 1.000 |
| rs2814184 | 10 | 133945696 | *JAKMIP3* | G/A | A | 2.19 | (1.4, 3.4) | 1.05 | (0.62, 1.77) | 1.07 | (0.77, 1.49) | 0.49 | (0.15, 0.82) | 4.14E-03 | 8.02E-03 | rs2637642 | 0.992 |
| rs2637642 | 10 | 133947657 | *JAKMIP3* | G/A | A | 2.26 | (1.45, 3.5) | 1.06 | (0.63, 1.79) | 1.05 | (0.75, 1.47) | 0.50 | (0.18, 0.83) | 2.15E-03 | 4.16E-03 | rs2637642 | 1.000 |
| rs2814181 | 10 | 133949032 | *JAKMIP3* | A/G | G | 2.17 | (1.39, 3.37) | 1.06 | (0.63, 1.78) | 1.07 | (0.77, 1.49) | 0.48 | (0.14, 0.82) | 5.27E-03 | 1.02E-02 | rs2637642 | 0.989 |
| rs75340217 | 11 | 114266216 | *C11orf71* | G/A | G | 2.50 | (1.34, 4.67) | 1.06 | (0.51, 2.2) | 1.18 | (0.87, 1.6) | 0.50 | (0.07, 0.94) | 2.29E-02 | 4.41E-02 | rs78532571 | 0.842 |
| rs76860109 | 11 | 114269160 | *C11orf71* | A/G | A | 2.50 | (1.34, 4.67) | 1.06 | (0.51, 2.2) | 1.18 | (0.87, 1.6) | 0.50 | (0.07, 0.94) | 2.29E-02 | 4.41E-02 | rs78532571 | 0.842 |
| rs79975601 | 11 | 114273064 | *RBM7* | A/G | A | 2.50 | (1.34, 4.67) | 1.06 | (0.51, 2.2) | 1.18 | (0.87, 1.6) | 0.50 | (0.07, 0.94) | 2.29E-02 | 4.41E-02 | rs78532571 | 0.842 |
| rs78226978 | 11 | 114275226 | *RBM7* | G/A | G | 2.50 | (1.34, 4.67) | 1.06 | (0.51, 2.2) | 1.18 | (0.87, 1.6) | 0.50 | (0.07, 0.94) | 2.29E-02 | 4.41E-02 | rs78532571 | 0.842 |
| rs75472325 | 11 | 114281162 | *1.5kb 3' of RBM7* | G/A | G | 2.55 | (1.36, 4.78) | 1.08 | (0.52, 2.25) | 1.20 | (0.88, 1.63) | 0.50 | (0.06, 0.93) | 2.57E-02 | 4.94E-02 | rs78532571 | 0.842 |
| rs79554856 | 11 | 114284418 | *4.8kb 3' of RBM7* | G/A | G | 2.50 | (1.34, 4.67) | 1.06 | (0.51, 2.2) | 1.18 | (0.87, 1.6) | 0.50 | (0.07, 0.94) | 2.29E-02 | 4.41E-02 | rs78532571 | 0.842 |
| rs78317528 | 11 | 114290009 | *10kb 3' of RBM7* | G/A | G | 2.50 | (1.34, 4.67) | 1.06 | (0.51, 2.2) | 1.18 | (0.87, 1.6) | 0.50 | (0.07, 0.94) | 2.29E-02 | 4.41E-02 | rs78532571 | 0.842 |
| rs78532571 | 11 | 114324733 | *3.7kb 3' of REXO2* | C/A | C | 2.81 | (1.49, 5.29) | 1.19 | (0.6, 2.37) | 1.19 | (0.87, 1.61) | 0.51 | (0.09, 0.93) | 1.64E-02 | 3.15E-02 | rs78532571 | 1.000 |
| rs77598260 | 11 | 114335779 | *15kb 3' of REXO2* | C/A | C | 2.69 | (1.44, 5.05) | 1.16 | (0.58, 2.3) | 1.19 | (0.87, 1.61) | 0.50 | (0.08, 0.92) | 2.06E-02 | 3.98E-02 | rs78532571 | 0.994 |
| rs34658647 | 11 | 128340977 | *ETS1* | C/A | C | 1.74 | (1.16, 2.61) | 1.02 | (0.66, 1.56) | 1.02 | (0.69, 1.49) | 0.41 | (0.06, 0.75) | 1.99E-02 | 3.83E-02 | rs34658647 | 1.000 |
| rs12575164 | 11 | 128349430 | *ETS1* | G/A | G | 1.74 | (1.16, 2.61) | 1.02 | (0.66, 1.56) | 1.02 | (0.69, 1.49) | 0.41 | (0.06, 0.75) | 2.01E-02 | 3.87E-02 | rs34658647 | 0.990 |
| rs7953094 | 12 | 75158784 | *224kb 3' of ATXN7L3B* | A/G | A | 2.53 | (1.38, 4.61) | 1.01 | (0.47, 2.16) | 1.17 | (0.86, 1.58) | 0.53 | (0.12, 0.95) | 1.11E-02 | 2.13E-02 | rs7965596 | 1.000 |
| rs7965596 | 12 | 75162157 | *227kb 3' of ATXN7L3B* | A/G | A | 2.53 | (1.39, 4.62) | 1.01 | (0.47, 2.16) | 1.16 | (0.86, 1.58) | 0.53 | (0.12, 0.95) | 1.10E-02 | 2.12E-02 | rs7965596 | 1.000 |
| **rs1570541** | 13 | 76074865 | *19kb 5' of TBC1D4* | G/A | G | 2.63 | (1.62, 4.29) | 1.09 | (0.6, 1.97) | 1.09 | (0.79, 1.5) | 0.55 | (0.23, 0.87) | 8.32E-04 | 1.61E-03 | rs6562898 | 1.000 |
| **rs6562898** | 13 | 76076368 | *20kb 5' of TBC1D4* | A/G | A | 2.64 | (1.62, 4.3) | 1.10 | (0.61, 1.99) | 1.07 | (0.78, 1.48) | 0.56 | (0.23, 0.88) | 7.48E-04 | 1.45E-03 | rs6562898 | 1.000 |
| rs696780 | 13 | 77641249 | *MYCBP2* | G/A | G | 2.51 | (1.54, 4.08) | 1.16 | (0.67, 2) | 1.13 | (0.82, 1.56) | 0.49 | (0.14, 0.83) | 5.61E-03 | 1.08E-02 | rs696780 | 1.000 |
| rs4905636 | 14 | 98455553 | *11kb 5' of C14orf64* | A/G | A | 1.91 | (1.22, 2.99) | 1.00 | (0.61, 1.66) | 1.10 | (0.79, 1.54) | 0.42 | (0.05, 0.79) | 2.54E-02 | 4.89E-02 | rs4905636 | 1.000 |
| rs934297 | 15 | 58752734 | *LIPC* | C/A | A | 2.11 | (1.35, 3.27) | 1.14 | (0.69, 1.88) | 1.10 | (0.78, 1.54) | 0.41 | (0.05, 0.77) | 2.57E-02 | 4.94E-02 | rs934297 | 1.000 |
| rs4788119 | 16 | 29019356 | *17kb 3' of LAT* | C/A | C | 1.92 | (1.29, 2.86) | 1.14 | (0.75, 1.75) | 1.00 | (0.68, 1.48) | 0.40 | (0.07, 0.73) | 1.63E-02 | 3.14E-02 | rs4788119 | 1.000 |
| **rs79128613** | 16 | 30788236 | *607bp 3' of RNF40* | G/A | G | 2.29 | (1.34, 3.91) | 1.04 | (0.57, 1.9) | 1.15 | (0.84, 1.57) | 0.48 | (0.09, 0.87) | 1.55E-02 | 2.98E-02 | rs79128613 | 0.972 |
| **rs78985803** | 16 | 30812608 | *14kb 5' of ZNF629* | A/G | A | 2.31 | (1.35, 3.95) | 1.03 | (0.56, 1.87) | 1.11 | (0.81, 1.52) | 0.51 | (0.14, 0.88) | 7.36E-03 | 1.42E-02 | rs78985803 | 1.000 |
| rs11642187 | 16 | 50578449 | *3.8kb 5' of NKD1* | C/A | C | 3.23 | (1.79, 5.81) | 1.52 | (0.82, 2.83) | 1.18 | (0.87, 1.62) | 0.47 | (0.06, 0.88) | 2.39E-02 | 4.60E-02 | rs11642187 | 1.000 |
| rs7186310 | 16 | 67305513 | *SLC9A5* | C/A | C | 2.01 | (1.28, 3.16) | 1.03 | (0.63, 1.69) | 1.11 | (0.8, 1.55) | 0.43 | (0.07, 0.79) | 1.78E-02 | 3.43E-02 | rs7186310 | 1.000 |
| rs11641114 | 16 | 83785148 | *CDH13* | A/G | A | 2.67 | (1.32, 5.41) | 1.01 | (0.5, 2.03) | 1.19 | (0.88, 1.61) | 0.55 | (0.14, 0.96) | 8.45E-03 | 1.63E-02 | rs11641114 | 1.000 |
| **rs2290065** | 17 | 38716360 | *CCR7* | A/G | A | 2.71 | (1.55, 4.75) | 1.12 | (0.59, 2.11) | 1.15 | (0.84, 1.57) | 0.53 | (0.17, 0.9) | 3.87E-03 | 7.49E-03 | rs2290065 | 1.000 |
| rs12953931 | 18 | 56724261 | *3.8kb 3' of LOC390858* | A/G | A | 1.69 | (1.13, 2.53) | 1.00 | (0.66, 1.53) | 1.01 | (0.68, 1.49) | 0.40 | (0.06, 0.75) | 2.21E-02 | 4.25E-02 | rs12953931 | 1.000 |
| rs526260 | 18 | 77553173 | *39kb 3' of CTDP1* | G/A | A | 2.18 | (1.39, 3.44) | 1.07 | (0.65, 1.74) | 1.09 | (0.78, 1.52) | 0.47 | (0.14, 0.8) | 5.23E-03 | 1.01E-02 | rs526260 | 1.000 |
| rs2916070 | 19 | 19524105 | *GATAD2A* | A/G | A | 1.93 | (1.26, 2.94) | 1.01 | (0.64, 1.6) | 1.05 | (0.74, 1.49) | 0.45 | (0.12, 0.78) | 7.48E-03 | 1.45E-02 | rs2916070 | 1.000 |
| rs889135 | 19 | 51141486 | *SYT3* | A/G | A | 2.49 | (1.48, 4.21) | 1.14 | (0.62, 2.08) | 1.14 | (0.83, 1.56) | 0.49 | (0.11, 0.86) | 1.08E-02 | 2.08E-02 | rs889135 | 1.000 |
| **rs73055288** | 19 | 55450462 | *NLRP7* | A/C | A | 2.75 | (1.59, 4.74) | 1.13 | (0.58, 2.19) | 1.14 | (0.83, 1.55) | 0.54 | (0.18, 0.9) | 3.56E-03 | 6.90E-03 | rs73055288 | 1.000 |
| rs760426 | 21 | 45715814 | *AIRE* | G/A | G | 2.04 | (1.3, 3.19) | 1.06 | (0.65, 1.75) | 1.10 | (0.79, 1.54) | 0.43 | (0.07, 0.79) | 1.99E-02 | 3.85E-02 | rs760426 | 1.000 |

**TABLE S4.** Summary of findings from gene-environment interaction between cigarette smoking in non-LS after adding adjustment for HLA-DRB1*03 at FDR < 0.05

| Marker name | CHR | Position (hg19) | Marker location w RefSeq Gene | Minor/Major alleles | coded allele | $OR_{SNP\text{-}SMOK}$ | 95%CI | $OR_{SNP}$ | 95%CI | $OR_{SMOK}$ | 95%CI | AP | 95%CI | AP p-val | FDR | Best tag SNP | LD $r^2$ |
|---|---|---|---|---|---|---|---|---|---|---|---|---|---|---|---|---|---|
| **rs12565567** | 1 | 67651412 | *IL23R* | A/G | A | 3.08 | (1.71, 5.56) | 1.21 | (0.63, 2.31) | 1.04 | (0.83, 1.3) | 0.59 | (0.25, 0.94) | 7.43E-04 | 1.69E-03 | rs61780312 | 0.840 |
| **rs17129698** | 1 | 67654072 | *IL23R* | A/G | A | 3.08 | (1.71, 5.56) | 1.23 | (0.64, 2.35) | 1.04 | (0.83, 1.31) | 0.59 | (0.24, 0.94) | 1.05E-03 | 2.39E-03 | rs61780312 | 0.842 |
| **rs61780310** | 1 | 67659353 | *IL23R* | G/A | G | 3.06 | (1.7, 5.51) | 1.20 | (0.63, 2.29) | 1.04 | (0.83, 1.31) | 0.59 | (0.25, 0.94) | 7.82E-04 | 1.78E-03 | rs61780312 | 0.852 |
| **rs61780311** | 1 | 67661307 | *IL23R* | G/C | G | 3.25 | (1.76, 6) | 1.22 | (0.62, 2.41) | 1.05 | (0.84, 1.31) | 0.61 | (0.26, 0.96) | 5.57E-04 | 1.27E-03 | rs61780312 | 0.989 |
| **rs61780312** | 1 | 67661648 | *IL23R* | C/A | C | 3.25 | (1.76, 5.99) | 1.19 | (0.61, 2.34) | 1.04 | (0.84, 1.31) | 0.62 | (0.28, 0.96) | 3.24E-04 | 7.40E-04 | rs61780312 | 1.000 |
| **rs28464018** | 1 | 67662430 | *IL23R* | G/A | G | 3.25 | (1.76, 5.99) | 1.20 | (0.61, 2.35) | 1.05 | (0.84, 1.31) | 0.62 | (0.28, 0.96) | 3.59E-04 | 8.20E-04 | rs61780312 | 0.994 |
| **rs116482055** | 1 | 101263357 | *59kb 3' of VCAM1* | A/G | A | 2.19 | (1.31, 3.66) | 1.10 | (0.63, 1.93) | 1.04 | (0.83, 1.31) | 0.48 | (0.09, 0.86) | 1.46E-02 | 3.30E-02 | rs116482055 | 1.000 |
| **rs7554873** | 1 | 161612233 | *11kb 5' of FCGR3B* | G/A | G | 1.98 | (1.28, 3.08) | 1.10 | (0.67, 1.8) | 1.03 | (0.81, 1.29) | 0.43 | (0.07, 0.8) | 2.05E-02 | 4.63E-02 | rs7554873 | 1.000 |
| **rs114384494** | 1 | 161653554 | *RPL31P11* | A/G | A | 2.13 | (1.34, 3.39) | 1.08 | (0.62, 1.88) | 1.03 | (0.82, 1.29) | 0.48 | (0.11, 0.85) | 1.13E-02 | 2.56E-02 | rs114384494 | 1.000 |
| **rs12760697** | 1 | 192416200 | *80kb 3' of RGS21* | A/G | A | 2.85 | (1.51, 5.37) | 1.30 | (0.67, 2.5) | 1.06 | (0.85, 1.32) | 0.52 | (0.1, 0.94) | 1.47E-02 | 3.32E-02 | rs12760697 | 1.000 |
| **rs410607** | 2 | 185445880 | *17kb 5' of ZNF804A* | A/G | A | 2.46 | (1.38, 4.38) | 1.09 | (0.54, 2.2) | 1.05 | (0.84, 1.31) | 0.54 | (0.13, 0.95) | 9.78E-03 | 2.22E-02 | rs410607 | 1.000 |
| **rs359900** | 2 | 185451906 | *11kb 5' of ZNF804A* | A/G | A | 2.46 | (1.38, 4.38) | 1.09 | (0.54, 2.2) | 1.05 | (0.84, 1.31) | 0.54 | (0.13, 0.95) | 9.78E-03 | 2.22E-02 | rs410607 | 1.000 |
| **rs359901** | 2 | 185452728 | *10kb 5' of ZNF804A* | A/G | A | 2.46 | (1.38, 4.38) | 1.09 | (0.54, 2.2) | 1.05 | (0.84, 1.31) | 0.54 | (0.13, 0.95) | 9.78E-03 | 2.22E-02 | rs410607 | 1.000 |
| **rs35272229** | 2 | 197946392 | *ANKRD44* | A/G | A | 1.83 | (1.17, 2.86) | 1.02 | (0.66, 1.58) | 1.03 | (0.82, 1.3) | 0.43 | (0.07, 0.78) | 1.78E-02 | 4.03E-02 | rs35272229 | 1.000 |
| **rs17245714** | 5 | 34998894 | *AGXT2* | C/G | C | 1.96 | (1.29, 2.96) | 1.08 | (0.69, 1.68) | 1.01 | (0.8, 1.28) | 0.44 | (0.11, 0.78) | 9.26E-03 | 2.11E-02 | rs17245714 | 1.000 |
| rs79850056 | 5 | 40513666 | *166kb 5' of PTGER4* | G/A | G | 2.62 | (1.44, 4.77) | 1.29 | (0.73, 2.29) | 1.06 | (0.84, 1.33) | 0.49 | (0.07, 0.9) | 2.08E-02 | 4.70E-02 | rs79850056 | 1.000 |
| **rs2106491** | 5 | 135348376 | *16kb 5' of TGFBI* | A/G | G | 2.25 | (1.3, 3.91) | 1.01 | (0.54, 1.88) | 1.04 | (0.83, 1.3) | 0.54 | (0.15, 0.92) | 5.81E-03 | 1.32E-02 | rs2106491 | 1.000 |
| **rs17658318** | 5 | 150322270 | *ZNF300P1* | A/G | A | 1.98 | (1.32, 2.98) | 1.06 | (0.69, 1.62) | 1.00 | (0.79, 1.27) | 0.46 | (0.15, 0.78) | 3.78E-03 | 8.62E-03 | rs17658318 | 1.000 |
| rs1133202 | 5 | 150489390 | *ANXA6* | A/G | G | 2.15 | (1.23, 3.75) | 1.04 | (0.55, 1.98) | 1.05 | (0.84, 1.31) | 0.49 | (0.07, 0.91) | 2.13E-02 | 4.81E-02 | rs1133202 | 1.000 |
| **rs10485038** | 6 | 90814274 | *BACH2* | G/C | G | 3.23 | (1.86, 5.6) | 1.51 | (0.87, 2.6) | 1.05 | (0.84, 1.31) | 0.52 | (0.16, 0.88) | 4.43E-03 | 1.01E-02 | rs57858238 | 0.995 |
| **rs57858238** | 6 | 90820248 | *BACH2* | A/G | A | 3.30 | (1.9, 5.74) | 1.51 | (0.87, 2.6) | 1.05 | (0.84, 1.31) | 0.53 | (0.18, 0.88) | 3.19E-03 | 7.27E-03 | rs57858238 | 1.000 |
| **rs10498966** | 6 | 91032094 | *9.5kb 3' of MIR4464* | G/A | G | 2.37 | (1.4, 4.01) | 1.03 | (0.58, 1.82) | 1.03 | (0.82, 1.29) | 0.55 | (0.21, 0.89) | 1.53E-03 | 3.49E-03 | rs10498966 | 1.000 |
| **rs62427027** | 6 | 126491161 | *131kb 3' of TRMT11* | A/G | A | 1.81 | (1.2, 2.73) | 1.05 | (0.68, 1.61) | 1.02 | (0.81, 1.29) | 0.41 | (0.06, 0.76) | 2.15E-02 | 4.87E-02 | rs62427027 | 1.000 |
| **rs2724099** | 7 | 93435670 | *80kb 3' of TFPI2* | G/A | G | 1.72 | (1.18, 2.51) | 1.03 | (0.67, 1.58) | 1.00 | (0.79, 1.27) | 0.40 | (0.06, 0.74) | 2.10E-02 | 4.74E-02 | rs2724099 | 1.000 |
| **rs61729556** | 11 | 7694002 | *CYB5R2* | A/G | A | 2.24 | (1.41, 3.56) | 1.10 | (0.61, 1.97) | 1.02 | (0.82, 1.29) | 0.50 | (0.13, 0.86) | 7.83E-03 | 1.78E-02 | rs61729556 | 1.000 |
| **rs79460410** | 11 | 60777914 | *CD6* | A/G | A | 2.77 | (1.45, 5.29) | 1.03 | (0.52, 2.05) | 1.05 | (0.84, 1.31) | 0.61 | (0.25, 0.97) | 8.95E-04 | 2.04E-03 | rs79460410 | 1.000 |
| rs1268594 | 14 | 59843837 | *7.4kb 3' of DAAM1* | G/A | G | 1.93 | (1.27, 2.94) | 1.11 | (0.71, 1.71) | 1.02 | (0.81, 1.29) | 0.42 | (0.07, 0.76) | 1.90E-02 | 4.31E-02 | rs1268594 | 1.000 |
| **rs177372** | 14 | 73757745 | *NUMB* | A/G | A | 2.48 | (1.27, 4.83) | 1.07 | (0.53, 2.14) | 1.06 | (0.85, 1.32) | 0.54 | (0.12, 0.97) | 1.22E-02 | 2.76E-02 | rs177372 | 1.000 |
| **rs177369** | 14 | 73760442 | *NUMB* | G/A | G | 2.45 | (1.26, 4.75) | 1.06 | (0.53, 2.12) | 1.06 | (0.85, 1.32) | 0.54 | (0.11, 0.97) | 1.29E-02 | 2.92E-02 | rs177372 | 0.988 |
| **rs5479** | 16 | 67469733 | *HSD11B2* | A/C | A | 2.34 | (1.4, 3.93) | 1.09 | (0.61, 1.96) | 1.04 | (0.83, 1.3) | 0.52 | (0.15, 0.88) | 5.54E-03 | 1.26E-02 | rs75204333 | 0.917 |
| **rs75204333** | 16 | 67574408 | *FAM65A* | G/A | G | 2.31 | (1.39, 3.83) | 1.04 | (0.58, 1.85) | 1.03 | (0.82, 1.29) | 0.54 | (0.19, 0.89) | 2.37E-03 | 5.41E-03 | rs75204333 | 1.000 |
| **rs4987801** | 18 | 60845099 | *BCL2* | G/A | G | 2.03 | (1.32, 3.14) | 1.13 | (0.71, 1.79) | 1.03 | (0.82, 1.3) | 0.43 | (0.08, 0.78) | 1.65E-02 | 3.74E-02 | rs4987801 | 1.000 |

**TABLE S5.** Summary of significant gene networks based on SNP-smoking interactions in LS and non-LS, respectively at FDR < 0.05

**LS SNP-SMOK interactions**

| # | Network | GO processes | Total nodes | Seed nodes | Pathways | p-Value | zScore | gScore |
|---|---|---|---|---|---|---|---|---|
| 1 | MAST4, TNIP1, SLIT3, RUNX3, ERK2 (MAPK1) | positive regulation of nitrogen compound metabolic process (87.2%; 6.364e-25), positive regulation of macromolecule biosynthetic process (74.5%; 1.227e-24), positive regulation of nucleobase-containing compound metabolic process (74.5%; 1.369e-24), positive regulation of cellular metabolic process (87.2%; 3.444e-24), positive regulation of macromolecule metabolic process (87.2%; 4.032e-24) | 50 | 4 | 0 | 1.610E-10 | 43.38 | 43.38 |
| 2 | RREB1, TNIP1, SP1, Norepinephrine + Na('+) + Cl('-) = Norepinephrine + Cl('-) + Na('+), Norepinephrine + H('+) = H('+) + Norepinephrine | regulation of gamma-aminobutyric acid secretion (20.0%; 7.534e-16), positive regulation of gamma-aminobutyric acid secretion (17.1%; 2.330e-14), feeding behavior (28.6%; 5.743e-14), adenylate cyclase-inhibiting G-protein coupled receptor signaling pathway (25.7%; 1.646e-13), positive regulation of neurotransmitter transport (22.9%; 1.885e-13) | 50 | 3 | 0 | 7.730E-08 | 33.18 | 33.18 |
| 3 | APG10, ADHFE1, CLIP170, CDK1 (p34), LBP9 | cell migration (50.0%; 6.711e-18), peptidyl-tyrosine autophosphorylation (20.8%; 8.394e-18), localization of cell (50.0%; 1.049e-16), cell motility (50.0%; 1.049e-16), locomotion (52.1%; 5.107e-16) | 50 | 3 | 0 | 8.760E-08 | 32.51 | 32.51 |
| 4 | MYCBP2, PA24A, SP1, PKC-theta, CDK2 | regulation of cell proliferation (67.5%; 3.010e-17), response to organonitrogen compound (60.0%; 8.927e-17), response to organic substance (82.5%; 1.268e-16), response to nitrogen compound (60.0%; 6.104e-16), response to hormone (57.5%; 7.984e-16) | 50 | 2 | 0 | 2.760E-05 | 22.82 | 22.82 |

**Non-LS SNP-SMOK interactions**

| # | Network | GO processes | Total nodes | Seed nodes | Pathways | p-Value | zScore | gScore |
|---|---|---|---|---|---|---|---|---|
| 1 | BACH2, NUMB, IL23R, CD6, G-protein alpha-q | positive regulation of cellular process (91.7%; 9.839e-19), positive regulation of biological process (91.7%; 1.487e-16), regulation of cell proliferation (60.4%; 1.747e-16), positive regulation of cell proliferation (50.0%; 2.025e-16), signaling (89.6%; 2.716e-16) | 50 | 4 | 0 | 2.770E-12 | 69.93 | 69.93 |
| 2 | Bcl-2, HSD11B2, ANKRD44, GCN5, DNA-PK | canonical Wnt signaling pathway (50.0%; 6.199e-32), regulation of animal organ morphogenesis (50.0%; 3.949e-24), non-canonical Wnt signaling pathway (42.1%; 1.066e-23), cell-cell signaling by wnt (52.6%; 1.098e-23), Wnt signaling pathway (52.6%; 1.098e-23) | 50 | 3 | 0 | 3.390E-09 | 54.83 | 54.83 |
